# Detection of Water Molecules on the Radical Transfer Pathway of Ribonucleotide Reductase by ^17^O Electron–Nuclear Double Resonance Spectroscopy

**DOI:** 10.1021/jacs.1c01359

**Published:** 2021-05-06

**Authors:** Fabian Hecker, JoAnne Stubbe, Marina Bennati

**Affiliations:** † Max Planck Institute for Biophysical Chemistry, 37077 Göttingen, Germany; ‡ Department of Chemistry, Massachusetts Institute of Technology, Cambridge, Massachusetts 20139, United States; § Department of Chemistry, Georg-August-University, 37077 Göttingen, Germany

## Abstract

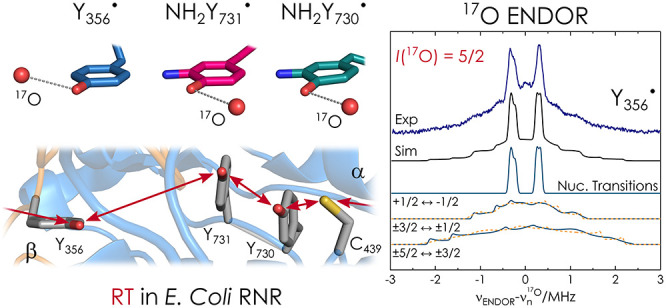

The role of water in biological proton-coupled electron transfer (PCET) is emerging as a key for understanding mechanistic details at atomic resolution. Here we demonstrate ^17^O high-frequency electron–nuclear double resonance (ENDOR) in conjunction with H_2_^17^O-labeled protein buffer to establish the presence of ordered water molecules at three radical intermediates in an active enzyme complex, the α_2_β_2_*E. coli* ribonucleotide reductase. Our data give unambiguous evidence that all three, individually trapped, intermediates are hyperfine coupled to one water molecule with Tyr-O···^17^O distances in the range 2.8–3.1 Å. The availability of this structural information will allow for quantitative models of PCET in this prototype enzyme. The results also provide a spectroscopic signature for water H-bonded to a tyrosyl radical.

Water is no longer known as just the solvent in which biochemical reactions take place but has been recognized as an essential player in these reactions.^1^ Of particular interest is water involvement in electron transfer processes,^2−5^ its action as a proton wire^6−8^ or its role in proton-coupled electron transfer (PCET).^9−12^ The identification of internal water in proteins can be achieved by X-ray diffraction.^13−15^ However, the crystallization of transient protein complexes is difficult. One key approach for detection of water in biological systems has been the use of ^17^O-enriched water in conjunction with magnetic resonance spectroscopy.^16−21^ Among these methods, electron paramagnetic resonance (EPR) can take advantage of high selectivity, as it detects nuclei only in the ligand sphere (*r* ≲ 1.5 nm)^22^ of paramagnetic centers.

EPR-based ^17^O hyperfine (hf) spectroscopy has been established for the detection of water binding to transition-metal ions, where the oxygen usually coordinates to the ion and large hyperfine couplings (several MHz) can be observed.^23−25^ However, the most common water coordination motif to biological radicals occurs via H-bond interactions. The hf coupling to ^17^O is diminished in comparison to the metal ion coordination, due to a longer interspin distance. In addition, the small ^17^O gyromagnetic ratio (γ_H_/γ_^17^O_ ≈ 7.4)^26^ and high nuclear spin (*I* = 5/2) have rendered the ^17^O hf coupling difficult to resolve.

Here we illustrate that high-frequency (94 and 263 GHz) electron–nuclear double resonance (ENDOR) spectroscopy can detect the ^17^O signal of ordered water molecules at an H-bond distance to radical intermediates in *E. coli* ribonucleotide reductase (RNR). The enzyme uses a long-range (32 Å) radical transfer (RT) to initiate nucleotide reduction (Scheme 1).^27^ Three tyrosines (Y_356_, Y_731_, and Y_730_) are essential pathway residues, which form transient intermediates in the active complex α_2_β_2_, consisting of the two homodimeric subunits α_2_ and β_2_.^13,27^ Water has been observed only crystallographically in inactive α_2_s without β_2_.^13,14,28,29^ Using site-selectively inserted tyrosine analogues to trap Y intermediates,^30^ our previous ^1^H/^2^H ENDOR and DFT studies^10,11,31^ revealed H-bonds attributed to water molecules and proposed a role of water in RT. However, all active sites of proteins have exchangeable protons, and thus alternative interpretations to our water proposal were possible. Recently, a cryo-EM structure of α_2_β_2_ was reported but the resolution was insufficient for water observation.^32^ Since our original proposals, studies using photo-RNRs and MD simulations implying waters in α_2_β_2_ have appeared.^33,34^ However, water has never been directly detected.

**Scheme 1:**
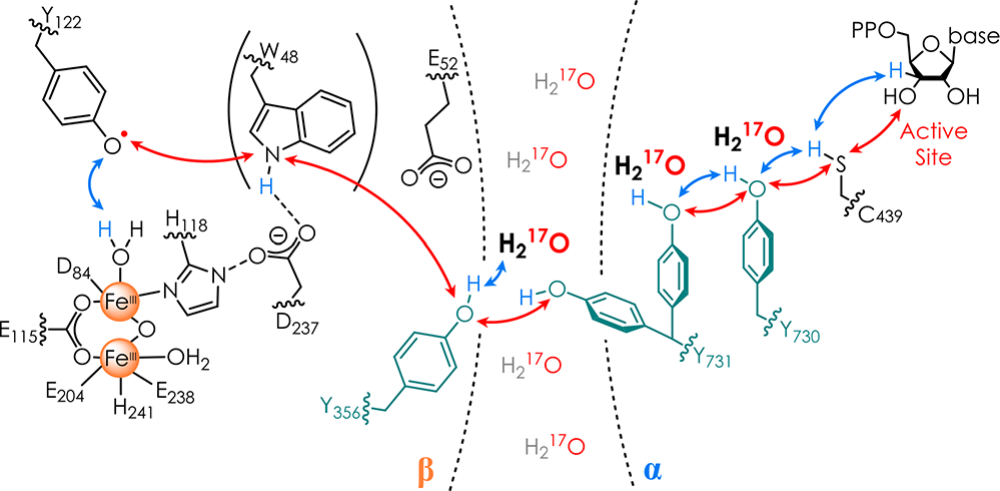
Current PCET Model of the 32 Å (Y_122_ to C_439_) RT in *E. coli* RNR^27,32^^,^ Redox-active tyrosines 356, 731, and 730 are shown in cyan, electron transfer steps as red arrows, and proton transfer steps as blue arrows. Water molecules revealed in this study in respective site-selective mutants are shown in boldface.

Therefore, we explored the capability of H_2_^17^O ENDOR spectroscopy by exchanging the RNR buffer with H_2_^17^O. α_2_β_2_-Y_356_^•^ was generated by a 2,3,5-F_3_Y_122_^•^ mutation in β_2_,^35^ whereas radicals at Y_731_ and Y_730_ were trapped by replacing the respective residue with 3-aminotyrosine (NH_2_Y),^36^ leading to α_2_β_2_-NH_2_Y_731_^•^ and α_2_β_2_-NH_2_Y_730_^•^. The individual variants were mixed with the complementary α_2_ or β_2_ protein, CDP as a substrate, and ATP as an effector. The reaction was then quenched after a few seconds inside EPR tubes. Details on the sample preparation are given in sections SI1 and SI2.

Figure 1 displays representative 94 GHz ^17^O Mims^37^ ENDOR spectra of the radical intermediates.

**Figure 1:**
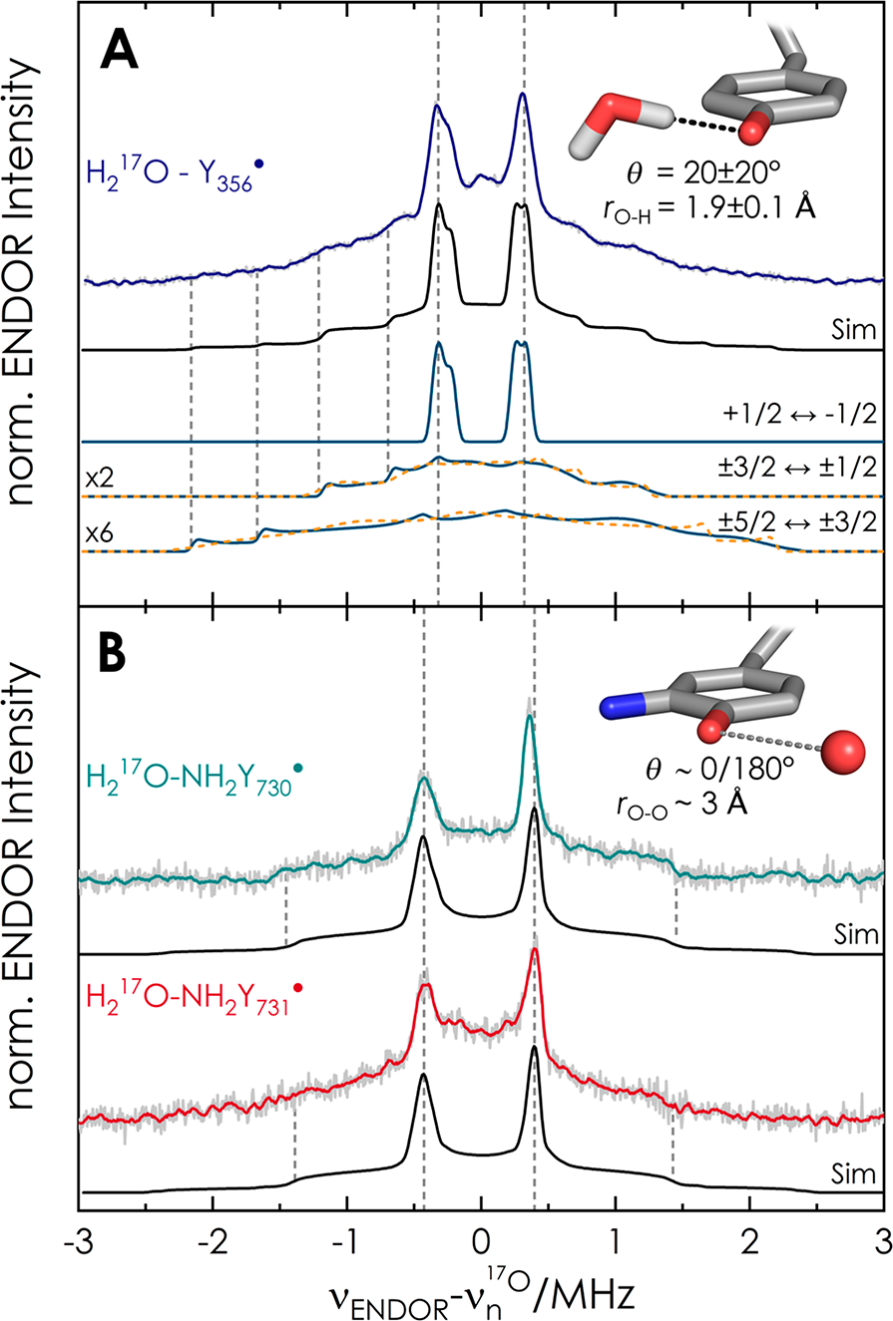
94 GHz ^17^O Mims^37^ ENDOR spectra of (A) the intermediate Y_356_^•^ and (B) NH_2_Y_731_^•^ and NH_2_Y_730_^•^ at *B*_0_∥*g*_*y*_ in the EPR line (*T* = 50 K, τ_Mims_ = 390 ns). Acquisition time: 46 h (Y_356_^•^), 40 h (NH_2_Y_731_^•^), and 18 h (NH_2_Y_730_^•^). Y_356_^•^ is from β_2_-F_3_Y_122_^•^/α_2_-Y_730_F, which gives the highest radical yield (section SI2). Experimental spectra are shown in gray, with a Savitzky–Golay filter (fourth-order polynomial, 20-point window) shown in color. Simulations used Easyspin^38^ (section SI1.6) with parameters given in Table 1 and section S3. Solid lines (teal) represent transitions among *m*_*I*_ > 0 manifolds and dashed lines (orange) those among *m*_*I*_ < 0 manifolds. The simulation does not distinguish between dihedral θ = 0°or θ = 180°.

Each spectrum shows a sharp doublet centered on the ^17^O Larmor frequency (19.3 MHz at 3.4 T), which can be assigned to the central spin transition (*m*_*I*_(^17^O) = +1/2 → −1/2) of one coupled ^17^O nucleus. As ^17^O is contained only in the water of the protein buffer, these sharp signals must arise from water molecules coupled to the radicals. Control experiments with only β_2_ protein confirmed that the signal is associated to the radicals generated in α_2_β_2_ (section SI3). The broad resonances at ±2.5 MHz are attributed to other nuclear transitions of the *I* = 5/2 spin system, broadened by nuclear quadrupole coupling (Figure 1A). Additionally, we note asymmetry of the doublet, which arises from second-order effects of the quadrupole coupling (section SI4). A comparison of the ENDOR spectra at the low (*B*_0_∥*g*_*x*_) and high-field (*B*_0_∥*g*_*z*_) edges of the EPR line (section SI5) indicates an almost isotropic hf coupling, with the dipolar contribution dominating the line width of the central doublet. The ^17^O ENDOR spectra could be simulated with one ^17^O nucleus, from which the asymmetry of the central peaks resulted using full diagonalization of the spin Hamiltonian (Figure 1 and section SI8). Parameters are given in Table 1 and section SI3. The spectra of Y_356_^•^ and NH_2_Y_731_^•^ additionally contain signals close to the Larmor frequency not reproduced in the simulations, which likely originate from second-sphere water molecules at the subunit interface. Additional broadening is also observed, particularly at NH_2_Y_731_^•^. It might be caused by conformational distribution of this residue, which was found to have flexibility.^39,40,33^

**Table 1:** Simulation and DFT Parameters for ^17^O and ^1^H hf Couplings of Water in RNR Intermediates^a^

	Y_356_^•^ sim/DFT_small_	NH_2_Y_731_^•^ sim	NH_2_Y_730_^•^ sim/DFT_large_^b^
*A*_*x*_ (^17^O)	0.43/0.19	0.70	0.65/0.24
*A*_*y*_ (^17^O)	0.66/0.59	0.84	0.80/0.6
*A*_*z*_ (^17^O)	0.70/0.65	0.89	0.89/0.6
*A*(H_1_)	6.2^31^/7.4	≲2.5^b^	2.7^b^/4.2
ρ(^17^O)^c^ (%)	0.05		0.03
*r*_O···^17^O_ (Å)	2.9 ± 0.1	∼3.0	∼3.0

a Except as noted, values are in MHz. Simulated quadrupole values for ^17^O were {*P*_*x*_;*P*_*y*_;*P*_*z*_} = {−0.02;–0.32;0.34} MHz with *e*^*2*^*qQ*/*h* = 6.8 MHz and η = 0.93.^41^

b Values from ^2^H couplings in refs (11 and 10) using γ_^1^H_/γ_^2^H_ ≈ 6.5.^26^

c Loewdin spin density^42^ from DFT. Uncertainties in coupling constants are less than 10% for simulations and up to 20% for DFT.

To rationalize the coupling, we began with a DFT-optimized small model (25 atoms, details in section SI1) of Y_356_^•^, as previous ENDOR spectra revealed ^1^H couplings consistent with one water at the H-bond distance *r*_O–H_ ≈ 1.8 Å.^31^ The ^17^O coupling from this model was *A*_max_(^17^O) ≈ 1 MHz, slightly exceeding the present experimental value of 0.6 ± 0.05 MHz. To optimize the model, we computed dihedral θ (C_3_–C_4_–O···H) and distance scans for ^17^O couplings, including the quadrupole tensor and the relative energies (section SI6). The DFT equilibrium distance always resulted in *r*_O–H_ ≈ 1.8 Å. We found that hf couplings and energies vary significantly with θ, while the quadrupole coupling is less affected (Figure S9A–C). *A*_xyz_ values of ≲1 MHz are found for θ in the range ≲±30° (or equivalently 150° ≲ θ ≲ 240°): i.e., close to the ring plane. Water coordination in the ring plane also results in minimal relative energies (Figure S9B). Importantly, predicted spin densities on ^17^O are <0.1% but are sufficient for producing a marked ^17^O isotropic splitting. The spin density transfer or spin polarization is likely related to the H-bond nature. A distance scan for the optimized dihedral of +20° predicts *A*_max_(^17^O) in the range 0.75–0.56 MHz (Figure S10A) for *r*_O–H_ ≈ 1.8–2.0 Å. Consideration of the DFT-predicted ^1^H couplings (Figure S10B) and comparison with the experimental values^31^ of ∼6.2 (H_1_) and ∼1.6 MHz (H_2_) indicates that the water is located at *r*_Tyr-O···^17^O_ = 2.9 ± 0.1 Å, corresponding to an *r*_O–H_ value of 1.9 ± 0.1 Å. Notably, the DFT-predicted dipolar coupling (*T*_||_ ≈ 0.3 MHz, section SI6) is consistent with the point-dipole model and the aforementioned broadening of the sharp peaks.

Analogous DFT calculations were performed on the isolated amino tyrosyl NH_2_Y^•^.^10,11,36^ We observed a trend for the ^17^O hf coupling in the dihedral and distance scans (section SI7) very similar to the Y^•^ model. The calculation predicts that *A*_iso_(^17^O) of NH_2_Y^•^ is slightly larger (10–15%) than that of Y^•^ at similar Tyr-O···^17^O distances and orientations, which could explain the experimental observation. The amino group introduces an asymmetry in the radical, and the energetically most favored water orientation is found at the opposite side of the amino group (Figure S11B). Nevertheless, this small model could not account simultaneously for the ^17^O and ^1^H couplings observed for these two intermediates (Figure S12). As noted in a previous *g*_*x*_ calculation,^10^ the coordination of the water molecule to NH_2_Y^•^s is influenced by the surrounding second-sphere residues, as these two intermediates are buried in α_2_β_2_ (Scheme 1).

Having established that at least one water molecule is hf-coupled to each of the three intermediates, we examine their current molecular models in light of this finding. First, we consider the radical site Y_356_^•^ (Scheme 1). To explain the unprecedented *g*_*x*_ value of Y_356_^•^ (*g*_*x*_ = 2.0062), we previously proposed that two almost equivalent waters might be simultaneously bonded to Y_356_^•^.^31^ While the present results are most consistent with the distance and orientation proposed for one water, the 94 GHz ^17^O ENDOR spectra (Figure 1A) cannot resolve a second water. We note that the spectral line shape and ^17^O hf coupling in Figure 1A are conserved in other RNR constructs that generate Y_356_^•^ (section SI8), including the F_3_Y_122_^•^/E_52_Q-β_2_ double mutant used to solve a recent cryo-EM structure.^43^

To gain spectral resolution, we recorded ^17^O ENDOR spectra of Y_356_^•^ at 263 GHz/9.4 T (Figure 2).^44−46^

**Figure 2:**
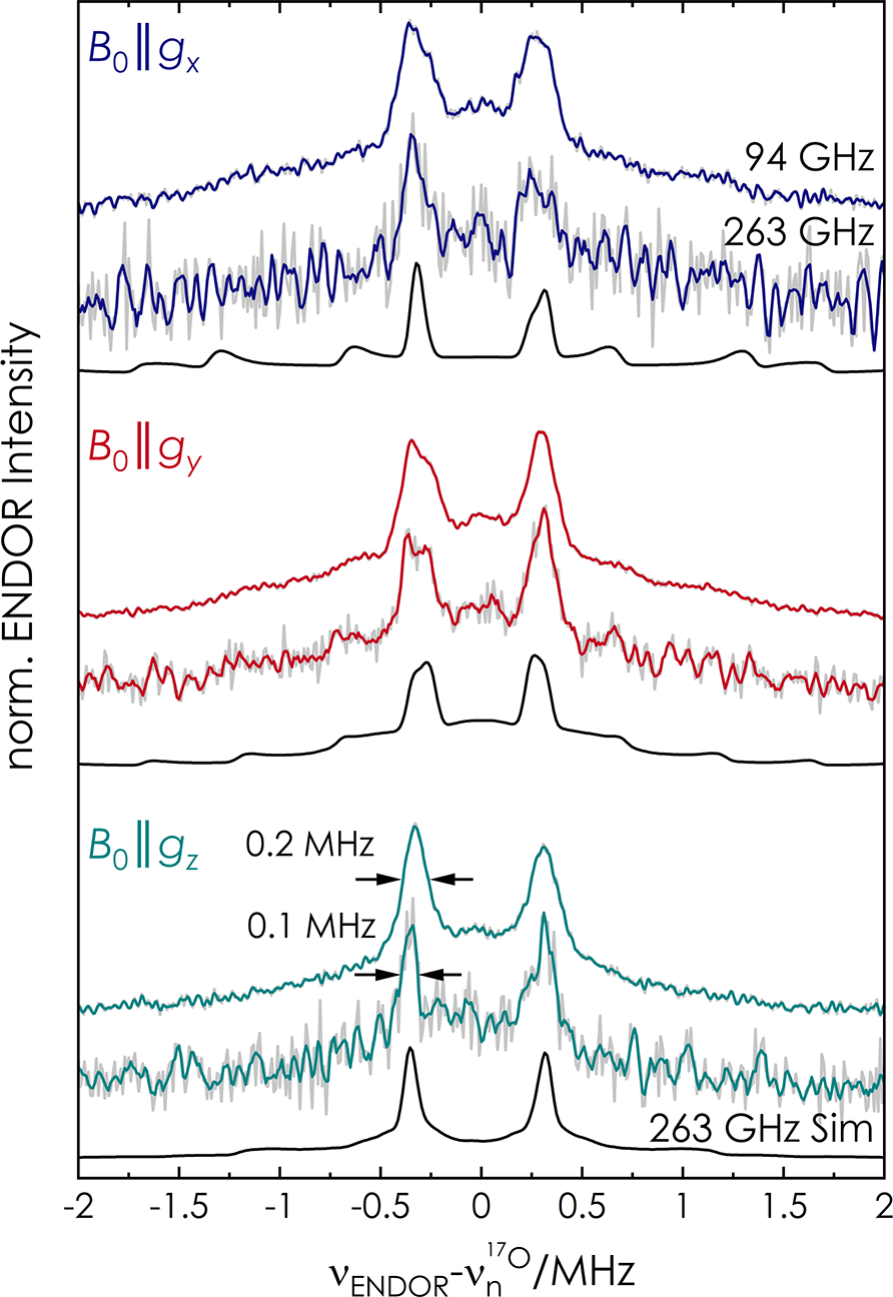
Comparison of 94 and 263 GHz Mims ENDOR of Y_356_^•^ at the three canonical positions in the EPR line. Total acquisition time for 263 GHz (*T* = 20 K): 18 h (*B*_0_∥*g*_*x*_), 10 h (*B*_0_∥*g*_*y*_), and 11 h (*B*_0_∥*g*_*z*_). Experimental spectra are shown in gray, with a Savitzky–Golay filter (fourth-order polynomial, 10 point window) in color. Simulations of 263 GHz spectra are in black with parameters as for 94 GHz (see Table 1 and Table S4).

The results illustrate that the line width of the central doublet substantially narrows, particularly at *B*_0_∥*g*_*z*_ (Figure 2). Despite the narrowing, a factor of approximately 2 from 94 to 263 GHz, we cannot discern two distinct ^17^O contributions. Simulations of the 263 GHz spectra with the same parameters used at 94 GHz reproduce the line narrowing and support the analysis at 94 GHz. The lack of evidence for a second, almost equivalent water H-bonded to Y_356_^•^ strongly suggests that the two-water model has become very unlikely and alternative explanations for the shifted *g*_*x*_ value of Y_356_^•^ will have to be examined. The precise location of second-sphere residues might play a role,^12^ which will require further experimental and computational investigation.

For the radical intermediates in the subunit α, a previous combined ENDOR/DFT model of NH_2_Y_730_^•^ proposed a water molecule coordinated in plane at a distance *r*_NH_2_Y_730_-O···^17^O_ ≈ 3.0 Å.^10^ The present results are consistent with this model and provide direct evidence for this postulated water in the enzyme complex α_2_β_2_-NH_2_Y_730_^•^. The DFT-predicted hf parameters (DFT_large_) for this large model (140 atoms) are reported in Table 1, and the model is displayed in section SI9.

Finally, for α_2_β_2_-NH_2_Y_731_^•^, large-scale (215 atoms) DFT calculations previously proposed three models of the trapped intermediate (section SI10). Among these models, only one (model 3, Figure S15) contained a water molecule at an H-bond distance. The DFT-predicted ^17^O hf couplings of model 3 (∼2.5 MHz), however, largely exceed the present experimental values (Table S5). However, this DFT model did not include residues from the β subunit, which we now know are close to this residue in the active complex.^43^ Therefore, the model will require further refinement. Nevertheless, the present results give evidence for a water molecule coordinated almost in the plane of NH_2_Y_731_^•^.

In conclusion, we have reported the capability of ^17^O high-frequency ENDOR to detect water H-bonded to tyrosyl radicals. The spectroscopic approach led to the first detection of ordered water molecules at three trapped radicals proposed to be representative of Y^•^ intermediates in the PCET of *E. coli* RNR. These results verify previous hypotheses on the presence and role of water in the RNR mechanism and provide a new starting point for computational studies. Knowledge of this ^17^O signature will also be generally useful for many other biological systems, in which tyrosyl radicals are involved.

## Supporting Information Available

Experimental procedure, radical yield determination, ENDOR spectra of Y_122_^•^ and F_3_Y_122_^•^, ENDOR spectra of *I* = 5/2 nuclei, orientation-selective ENDOR spectra, DFT models of Y^•^ and NH_2_Y^•^, ^17^O Y_356_^•^ spectra of different mutants, previous large DFT models of α_2_β_2_-NH_2_Y_730_^•^ and α_2_β_2_-NH_2_Y_731_^•^ (PDF)

## References

[ref1] Ball P. Water as an active constituent in cell biology. Chem. Rev. 2008, 108 (1), 74–108. 10.1021/cr068037a. 18095715

[ref2] Tezcan F. A.; Crane B. R.; Winkler J. R.; Gray H. B. Electron tunneling in protein crystals. Proc. Natl. Acad. Sci. U. S. A. 2001, 98 (9), 5002–6. 10.1073/pnas.081072898. 11296248 PMC33153

[ref3] van Amsterdam I. M.; Ubbink M.; Einsle O.; Messerschmidt A.; Merli A.; Cavazzini D.; Rossi G. L.; Canters G. W. Dramatic modulation of electron transfer in protein complexes by crosslinking. Nat. Struct. Biol. 2002, 9 (1), 48–52. 10.1038/nsb736. 11740504

[ref4] Lin J.; Balabin I. A.; Beratan D. N. The nature of aqueous tunneling pathways between electron-transfer proteins. Science 2005, 310 (5752), 1311–1313. 10.1126/science.1118316. 16311331 PMC3613566

[ref5] de la Lande A.; Marti S.; Parisel O.; Moliner V. Long distance electron-transfer mechanism in peptidylglycine alpha-hydroxylating monooxygenase: a perfect fitting for a water bridge. J. Am. Chem. Soc. 2007, 129 (38), 11700–7. 10.1021/ja070329l. 17764178

[ref6] Luecke H.; Schobert B.; Richter H. T.; Cartailler J. P.; Lanyi J. K. Structure of bacteriorhodopsin at 1.55 A resolution. J. Mol. Biol. 1999, 291 (4), 899–911. 10.1006/jmbi.1999.3027. 10452895

[ref7] Sass H. J.; Buldt G.; Gessenich R.; Hehn D.; Neff D.; Schlesinger R.; Berendzen J.; Ormos P. Structural alterations for proton translocation in the M state of wild-type bacteriorhodopsin. Nature 2000, 406 (6796), 649–53. 10.1038/35020607. 10949308

[ref8] Linke K.; Ho F. M. Water in Photosystem II: structural, functional and mechanistic considerations. Biochim. Biophys. Acta, Bioenerg. 2014, 1837 (1), 14–32. 10.1016/j.bbabio.2013.08.003. 23978393

[ref9] Saito K.; Shen J. R.; Ishida T.; Ishikita H. Short hydrogen bond between redox-active tyrosine Y(Z) and D1-His190 in the photosystem II crystal structure. Biochemistry 2011, 50 (45), 9836–44. 10.1021/bi201366j. 21972783

[ref10] Argirevic T.; Riplinger C.; Stubbe J.; Neese F.; Bennati M. ENDOR spectroscopy and DFT calculations: evidence for the hydrogen-bond network within α2 in the PCET of E. coli ribonucleotide reductase. J. Am. Chem. Soc. 2012, 134 (42), 17661–70. 10.1021/ja3071682. 23072506 PMC4516058

[ref11] Nick T. U.; Lee W.; Kossmann S.; Neese F.; Stubbe J.; Bennati M. Hydrogen bond network between amino acid radical intermediates on the proton-coupled electron transfer pathway of E. coli α2 ribonucleotide reductase. J. Am. Chem. Soc. 2015, 137 (1), 289–98. 10.1021/ja510513z. 25516424 PMC4304443

[ref12] Sirohiwal A.; Neese F.; Pantazis D. A. Microsolvation of the Redox-Active Tyrosine-D in Photosystem II: Correlation of Energetics with EPR Spectroscopy and Oxidation-Induced Proton Transfer. J. Am. Chem. Soc. 2019, 141 (7), 3217–3231. 10.1021/jacs.8b13123. 30666866 PMC6728127

[ref13] Uhlin U.; Eklund H. Structure of ribonucleotide reductase protein R1. Nature 1994, 370 (6490), 533–9. 10.1038/370533a0. 8052308

[ref14] Eriksson M.; Uhlin U.; Ramaswamy S.; Ekberg M.; Regnström K.; Sjöberg B.-M.; Eklund H. Binding of allosteric effectors to ribonucleotide reductase protein R1: reduction of active-site cysteines promotes substrate binding. Structure 1997, 5 (8), 1077–1092. 10.1016/S0969-2126(97)00259-1. 9309223

[ref15] Umena Y.; Kawakami K.; Shen J. R.; Kamiya N. Crystal structure of oxygen-evolving photosystem II at a resolution of 1.9 A. Nature 2011, 473 (7345), 55–60. 10.1038/nature09913. 21499260

[ref16] Schmidt B.; McCracken J.; Ferguson-Miller S. A discrete water exit pathway in the membrane protein cytochrome c oxidase. Proc. Natl. Acad. Sci. U. S. A. 2003, 100 (26), 15539–42. 10.1073/pnas.2633243100. 14660787 PMC307603

[ref17] Bennati M.; Hertel M. M.; Fritscher J.; Prisner T. F.; Weiden N.; Hofweber R.; Sporner M.; Horn G.; Kalbitzer H. R. High-frequency 94 GHz ENDOR characterization of the metal binding site in wild-type Ras x GDP and its oncogenic mutant G12V in frozen solution. Biochemistry 2006, 45 (1), 42–50. 10.1021/bi051156k. 16388579

[ref18] Potapov A.; Goldfarb D. The Mn(2+)-bicarbonate complex in a frozen solution revisited by pulse W-band ENDOR. Inorg. Chem. 2008, 47 (22), 10491–8. 10.1021/ic8011316. 18947176

[ref19] McConnell I. L.; Grigoryants V. M.; Scholes C. P.; Myers W. K.; Chen P. Y.; Whittaker J. W.; Brudvig G. W. EPR-ENDOR characterization of (^17^O, ^1^H, ^2^H) water in manganese catalase and its relevance to the oxygen-evolving complex of photosystem II. J. Am. Chem. Soc. 2012, 134 (3), 1504–12. 10.1021/ja203465y. 22142421 PMC3471538

[ref20] Nalepa A.; Malferrari M.; Lubitz W.; Venturoli G.; Mobius K.; Savitsky A. Local water sensing: water exchange in bacterial photosynthetic reaction centers embedded in a trehalose glass studied using multiresonance EPR. Phys. Chem. Chem. Phys. 2017, 19 (41), 28388–28400. 10.1039/C7CP03942E. 29034914

[ref21] Rowlands L. J.; Marks A.; Sanderson J. M.; Law R. V. ^17^O NMR spectroscopy as a tool to study hydrogen bonding of cholesterol in lipid bilayers. Chem. Commun. 2020, 56 (92), 14499–14502. 10.1039/D0CC05466F. 33150883

[ref22] Meyer A.; Dechert S.; Dey S.; Hobartner C.; Bennati M. Measurement of Angstrom to Nanometer Molecular Distances with ^19^F Nuclear Spins by EPR/ENDOR Spectroscopy. Angew. Chem., Int. Ed. 2020, 59 (1), 373–379. 10.1002/anie.201908584. PMC6973229 31539187

[ref23] Raitsimring A. M.; Astashkin A. V.; Baute D.; Goldfarb D.; Caravan P. W-Band ^17^O Pulsed Electron-Nuclear Double Resonance Study of Gadolinium Complexes with Water. J. Phys. Chem. A 2004, 108 (35), 7318–7323. 10.1021/jp040306i.

[ref24] Baute D.; Goldfarb D. The 17O hyperfine interaction in V^17^O(H_2_^17^O)^52+^ and Mn(H_2_^17^O)^62+^ determined by high field ENDOR aided by DFT calculations. J. Phys. Chem. A 2005, 109 (35), 7865–71. 10.1021/jp052132q. 16834167

[ref25] Rapatskiy L.; Cox N.; Savitsky A.; Ames W. M.; Sander J.; Nowaczyk M. M.; Rogner M.; Boussac A.; Neese F.; Messinger J.; Lubitz W. Detection of the water-binding sites of the oxygen-evolving complex of Photosystem II using W-band ^17^O electron-electron double resonance-detected NMR spectroscopy. J. Am. Chem. Soc. 2012, 134 (40), 16619–34. 10.1021/ja3053267. 22937979

[ref26] Tiesinga E.; Mohr P. J.; Newell D. B.; Taylor B. N. CODATA Recommended Values of the Fundamental Physical Constants: 2018; https://physics.nist.gov/cuu/Constants/index.html (accessed 2021-01-15). 10.1063/5.0064853 PMC9888147 36726646

[ref27] Greene B. L.; Kang G.; Cui C.; Bennati M.; Nocera D. G.; Drennan C. L.; Stubbe J. Ribonucleotide Reductases: Structure, Chemistry, and Metabolism Suggest New Therapeutic Targets. Annu. Rev. Biochem. 2020, 89, 45–75. 10.1146/annurev-biochem-013118-111843. 32569524 PMC7316142

[ref28] Yokoyama K.; Uhlin U.; Stubbe J. Site-specific incorporation of 3-nitrotyrosine as a probe of pKa perturbation of redox-active tyrosines in ribonucleotide reductase. J. Am. Chem. Soc. 2010, 132 (24), 8385–97. 10.1021/ja101097p. 20518462 PMC2905227

[ref29] Minnihan E. C.; Seyedsayamdost M. R.; Uhlin U.; Stubbe J. Kinetics of radical intermediate formation and deoxynucleotide production in 3-aminotyrosine-substituted Escherichia coli ribonucleotide reductases. J. Am. Chem. Soc. 2011, 133 (24), 9430–40. 10.1021/ja201640n. 21612216 PMC3125130

[ref30] Minnihan E. C.; Nocera D. G.; Stubbe J. Reversible, long-range radical transfer in E. coli class Ia ribonucleotide reductase. Acc. Chem. Res. 2013, 46 (11), 2524–35. 10.1021/ar4000407. 23730940 PMC3823682

[ref31] Nick T. U.; Ravichandran K. R.; Stubbe J.; Kasanmascheff M.; Bennati M. Spectroscopic Evidence for a H Bond Network at Y_356_ Located at the Subunit Interface of Active E. coli Ribonucleotide Reductase. Biochemistry 2017, 56 (28), 3647–3656. 10.1021/acs.biochem.7b00462. 28640584

[ref32] Kang G.; Taguchi A. T.; Stubbe J.; Drennan C. L. Structure of a trapped radical transfer pathway within a ribonucleotide reductase holocomplex. Science 2020, 368 (6489), 424–427. 10.1126/science.aba6794. 32217749 PMC7774503

[ref33] Reinhardt C. R.; Li P.; Kang G.; Stubbe J.; Drennan C. L.; Hammes-Schiffer S. Conformational Motions and Water Networks at the alpha/beta Interface in E. coli Ribonucleotide Reductase. J. Am. Chem. Soc. 2020, 142 (32), 13768–13778. 10.1021/jacs.0c04325. 32631052 PMC7594210

[ref34] Cui C.; Greene B. L.; Kang G.; Drennan C. L.; Stubbe J.; Nocera D. G. Gated Proton Release during Radical Transfer at the Subunit Interface of Ribonucleotide Reductase. J. Am. Chem. Soc. 2021, 143 (1), 176–183. 10.1021/jacs.0c07879. 33353307 PMC7904477

[ref35] Minnihan E. C.; Young D. D.; Schultz P. G.; Stubbe J. Incorporation of fluorotyrosines into ribonucleotide reductase using an evolved, polyspecific aminoacyl-tRNA synthetase. J. Am. Chem. Soc. 2011, 133 (40), 15942–5. 10.1021/ja207719f. 21913683 PMC3188361

[ref36] Lee W.; Kasanmascheff M.; Huynh M.; Quartararo A.; Costentin C.; Bejenke I.; Nocera D. G.; Bennati M.; Tommos C.; Stubbe J. Properties of Site-Specifically Incorporated 3-Aminotyrosine in Proteins To Study Redox-Active Tyrosines: Escherichia coli Ribonucleotide Reductase as a Paradigm. Biochemistry 2018, 57 (24), 3402–3415. 10.1021/acs.biochem.8b00160. 29630358 PMC6110390

[ref37] Mims W. B. Pulsed endor experiments. Proc. R. Soc. London, Ser. A 1965, 283 (1395), 452–457. 10.1098/rspa.1965.0034.

[ref38] Stoll S.; Schweiger A. EasySpin, a comprehensive software package for spectral simulation and analysis in EPR. J. Magn. Reson. 2006, 178 (1), 42–55. 10.1016/j.jmr.2005.08.013. 16188474

[ref39] Kasanmascheff M.; Lee W.; Nick T. U.; Stubbe J.; Bennati M. Radical transfer in E. coli ribonucleotide reductase: a NH_2_Y_731_/R411A-α mutant unmasks a new conformation of the pathway residue 731. Chem. Sci. 2016, 7 (3), 2170–2178. 10.1039/C5SC03460D. 29899944 PMC5968753

[ref40] Greene B. L.; Taguchi A. T.; Stubbe J.; Nocera D. G. Conformationally Dynamic Radical Transfer within Ribonucleotide Reductase. J. Am. Chem. Soc. 2017, 139 (46), 16657–16665. 10.1021/jacs.7b08192. 29037038 PMC5702266

[ref41] Edmonds D. T.; Zussman A. Pure quadrupole resonance of ^17^O in ice. Phys. Lett. A 1972, 41 (2), 167–169. 10.1016/0375-9601(72)91097-3.

[ref42] Löwdin P. O. On the Non-Orthogonality Problem Connected with the Use of Atomic Wave Functions in the Theory of Molecules and Crystals. J. Chem. Phys. 1950, 18 (3), 365–375. 10.1063/1.1747632.

[ref43] Lin Q.; Parker M. J.; Taguchi A. T.; Ravichandran K.; Kim A.; Kang G.; Shao J.; Drennan C. L.; Stubbe J. Glutamate 52-β at the α/β subunit interface of Escherichia coli class Ia ribonucleotide reductase is essential for conformational gating of radical transfer. J. Biol. Chem. 2017, 292 (22), 9229–9239. 10.1074/jbc.M117.783092. 28377505 PMC5454104

[ref44] Jakobsen H. J.; Bildsøe H.; Brorson M.; Wu G.; Gor’kov P. L.; Gan Z.; Hung I. High-Field ^17^O MAS NMR Reveals ^1^J(^17^O-^127^I) with its Sign and the NMR Crystallography of the Scheelite Structures for NaIO_4_ and KIO_4_. J. Phys. Chem. C 2015, 119 (25), 14434–14442. 10.1021/acs.jpcc.5b03721.

[ref45] Keeler E. G.; Michaelis V. K.; Colvin M. T.; Hung I.; Gor’kov P. L.; Cross T. A.; Gan Z.; Griffin R. G. ^17^O MAS NMR Correlation Spectroscopy at High Magnetic Fields. J. Am. Chem. Soc. 2017, 139 (49), 17953–17963. 10.1021/jacs.7b08989. 29111706 PMC8256432

[ref46] Tkach I.; Bejenke I.; Hecker F.; Kehl A.; Kasanmascheff M.; Gromov I.; Prisecaru I.; Hofer P.; Hiller M.; Bennati M. ^1^H high field electron-nuclear double resonance spectroscopy at 263 GHz/9.4T. J. Magn. Reson. 2019, 303, 17–27. 10.1016/j.jmr.2019.04.001. 30991287

