# Supplementary material for: Detection of Water Molecules on the Radical Transfer Pathway of Ribonucleotide Reductase by 17O Electron–Nuclear Double Resonance Spectroscopy
Source: J Am Chem Soc. 2021 May 6;143(19):7237–41. doi: 10.1021/jacs.1c01359 (PMC8154519; doi:10.1021/jacs.1c01359)
Supplement: Supplementary file 1 — ja1c01359_si_001.pdf [file ja1c01359_si_001.pdf]

Supplementary Information

Detection of Water Molecules on the Radical Transfer Pathway of Ribonucleotide Reductase by ^{17}O Electron-Nuclear Double Resonance Spectroscopy

Fabian Hecker[1], JoAnne Stubbe[2] and Marina Bennati[1,3,*]

[1]Max Planck Institute for Biophysical Chemistry, 37077 Göttingen, Germany.

[2]Department of Chemistry, Massachusetts Institute of Technology, 20139 Cambridge, USA.

[3]Department of Chemistry Georg-August-University, 37077 Göttingen, Germany.

Table of Contents

SI1 Experimental procedure ... 2

SI1.1 Sample preparation ... 2

SI1.2 EPR spectroscopy ... 3

SI1.3 Relaxation measurements ... 3

SI1.4 ENDOR spectroscopy ... 5

SI1.5 Choice of τ – value for Mims ENDOR ... 6

SI1.6 Simulation of ENDOR spectra ... 7

SI1.7 DFT calculations ... 8

SI2 Radical yield determination ... 9

SI3 Mims ENDOR spectra of Y_{122}• and F_3Y_{122}• ... 10

SI4 ENDOR spectra of I=5/2 nuclei ... 11

SI5 Orientation selective Mims ^{17}O ENDOR spectra of the radical intermediates ... 14

SI6 DFT models of an isolated tyrosyl radical with a coordinated water molecule ... 16

SI7 DFT models of an isolated amino tyrosyl radical with a coordinated water molecule ... 19

SI8 ^{17}O ENDOR of different mutants to generate Y_{356}• ... 21

SI9 Previous large DFT model of $\alpha 2\beta 2$–NH_2Y_{730}• ... 23

SI10 Previous large DFT models of $\alpha 2\beta 2$-NH_2Y_{731}• ... 24

REFERENCES ... 25

SI1 Experimental procedure

SI1.1 Sample preparation

90 % ^{17}O labelled water was purchased from Sigma Aldrich. The incorporation of unnatural amino acids into *E. coli* ribonucleotide reductase followed the previously reported protocols.[1-2] Purified α_2 (wild-type, $Y_{730}F$, NH_2Y_{731} and NH_2Y_{730}) was exchanged into 5 mM HEPES buffer (pH 7.6) containing 1.5 mM $MgSO_4$, 0.1 mM EDTA and 1 mM β-mercaptoethanol with Amicon spin filters (30 000 NMWL). 100 μL protein solution was supplemented with 300 μL buffer and spun at 12 000 g for 5 min. This process was repeated 6 times. ATP and CDP were added and the protein concentration was adjusted with assay buffer (50 mM HEPES pH 7.6, 15 mM $MgSO_4$, 1 mM EDTA) to yield a final concentration of 30 μM α_2, 500 μM ATP and 167 μM CDP. 100 μL quantities of this solution were frozen in liquid nitrogen and lyophilized overnight. The samples were rehydrated in 10 μL $H_2{}^{17}O$ to yield solutions of 300 μM α_2, 5 mM ATP and 1.67 mM CDP in assay buffer. Recovery of wild-type (wt) α_2 activity after the lyophilization procedure was checked by spectrophotometric activity assay and found to be 90 – 100 % (data not shown). Purified β_2 (wt, F_3Y_{122}, $F_3Y_{122}/E_{52}Q$) was exchanged into assay buffer with the abovementioned protocol and had the following concentrations: 890 μM wt-β_2, 980 μM F_3Y_{122}-β_2, 1600 μM $F_3Y_{122}/E_{52}Q$-β_2. EPR samples were prepared by mixing the previously described α_2 solutions containing substrate and effector with the corresponding β_2 solution (Table S1) and addition of $H_2{}^{17}O$ to a final concentration of 180 μM $\alpha_2\beta_2$, 3 mM ATP and 1 mM CDP. The final amount of $H_2{}^{17}O$ was approx. 80 %. The reaction mixtures were hand quenched in liq. N_2 inside EPR tubes. The quench times followed the previously established protocols for maximum radical yield.[3-5] EPR samples containing only β_2 with $H_2{}^{17}O$ were prepared by diluting the abovementioned solutions of β_2 (wt and F_3Y_{122}) with $H_2{}^{17}O$ to a final protein concentration of 180 μM and approx. 90 % $H_2{}^{17}O$. The β_2 solution was left to incubate for 10 min at 4°C to allow for sufficient exchange of water molecules within the protein, i.e. close to Y_{122}, and subsequently frozen in liq. N_2 inside the EPR tubes. W-Band samples contained 2 μL protein mixture in 0.9 mm OD/0.5 mm ID suprasil tubes. 263 GHz samples contained 30 – 50 nL protein mixture in 0.33 mm OD/0.2mm ID suprasil capillaries.

Table S1: Subunit combinations, quench times and radical yields.

α_2-subunit	β_2-subunit	Radical	Quench Time	Radical Yield[(a)]
$Y_{730}F$	2,3,5-F_3Y_{122}	$Y_{356}^\bullet$	10 - 20 s	40 %
wt	2,3,5-F_3Y_{122}	$Y_{356}^\bullet$	10 - 20 s	25 %
$Y_{730}F$	2,3,5-$F_3Y_{122}/E_{52}Q$	$Y_{356}^\bullet$	120 s	40 %
wt	2,3,5-$F_3Y_{122}/E_{52}Q$	$Y_{356}^\bullet$	120 s	35 %
F_2Y_{731}	2,3,5-$F_3Y_{122}/E_{52}Q$	$Y_{356}^\bullet$	20 s	5 %
NH_2Y_{731}	wt	$NH_2Y_{731}^\bullet$	10 - 20 s	10 %
NH_2Y_{730}	wt	$NH_2Y_{730}^\bullet$	10 - 20 s	30 %
-	wt	$Y_{122}^\bullet$	10 min	100 %
-	2,3,5-F_3Y_{122}	2,3,5-$F_3Y_{122}^\bullet$	10 min	100 %

(a) Method for radical determination is explained under section SI 2.

SI1.2 EPR spectroscopy

3.4 T EPR experiments were performed on a Bruker E680 pulsed W-band spectrometer with 2 W microwave power output. The optimal pulse length was determined by a Rabi nutation to 8-10 ns for a $\pi/2$ pulse at maximum output power. Echo detected EPR spectra for radical yield determination were recorded with a Hahn echo pulse sequence ($\pi/2$ - τ - π - τ - echo) with τ = 290 ns. Shot repetition time (SRT) and shots/point varied for different temperatures and radicals and are given in the figure captions.
9.4 T experiments were performed on a Bruker E780 pulsed 263 GHz quasi-optical spectrometer with 100 mW microwave power output. The optimal pulse length was determined by a Rabi nutation to 30 – 32 ns for a $\pi/2$ pulse at maximum output power.

SI1.3 Relaxation measurements

To optimize ENDOR experiments at 50 K, we measured the relaxation properties of each radical. All relaxation experiments were recorded at the maximum of the EPR line, i.e. $B_0 \| g_y$. The electron spin-lattice relaxation time (T_{1e}) was determined via an inversion recovery experiment (π - t - $\pi/2$ - τ - π - τ – echo, inset Fig S1). A bi-exponential fit (Eq. 1) to the echo intensity as a function of the time-interval t yielded T_{1e} as the largest time constant, while the smaller time constant was assigned to spectral diffusion. T_{1e} as the longest relaxation time determines the shot repetition time (SRT) of all experiments should be at least 2-3 times longer than T_{1e}.

$$I = I_{0,1}\exp\left(-\frac{t}{T_{1e}}\right) + I_{0,2}\exp\left(-\frac{t}{T_{SD}}\right) + C \qquad \text{with } T_{1e} > T_{SD} \ (1)$$

At 50 K, T_{1e} is 1.6 ms for the tyrosyl radical $Y_{356}^{\bullet}/Y_{730}F$-$\alpha$ and 2.9 ms and 4.6 ms for the two amino-tyrosyl radicals $NH_2Y_{731}^{\bullet}$ and $NH_2Y_{730}^{\bullet}$, respectively. Therefore all 50 K ENDOR experiments of $Y_{356}^{\bullet}$ were performed with 5 ms SRT, while 10 ms SRT was used for the two amino tyrosyl radicals.

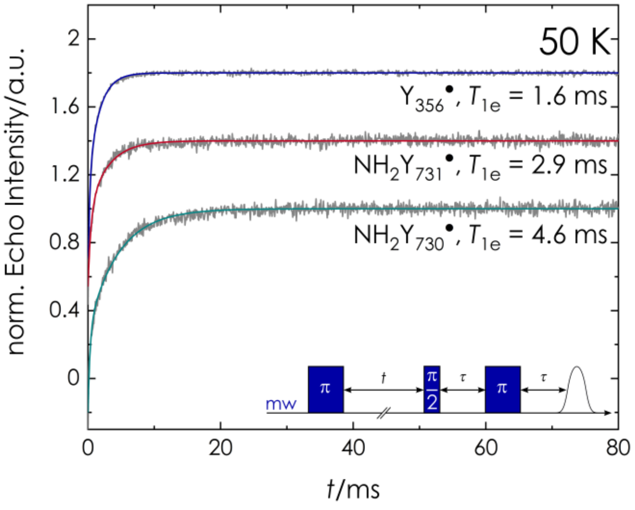

Figure S1: 50 K inversion recovery experiments of the three radical intermediates $Y_{356}^{\bullet}/Y_{730}F$-$\alpha$ (top, blue), $NH_2Y_{731}^{\bullet}$ (middle, red) and $NH_2Y_{730}^{\bullet}$ (bottom, cyan) in gray and bi exponential fits (Equation 1) in color. Largest time constant are given in the figure. Inset: microwave pulse sequence.

The phase memory time T_m strongly influences the Mims ENDOR sensitivity (see SI1.5). It was measured by recording the stimulated echo intensity as a function of the time delay τ (π/2 - τ - π/2 - T - π/2 - τ – echo, inset Fig. S2). T_m is the time constant of a mono exponential decay fit to the experimental data (data not shown):

$$I = I_0 \exp\left(-\frac{2\tau}{T_m}\right) + C \quad (2)$$

This experiment was repeated for increasing times T, i.e. the separation of the second and third π/2 pulse.

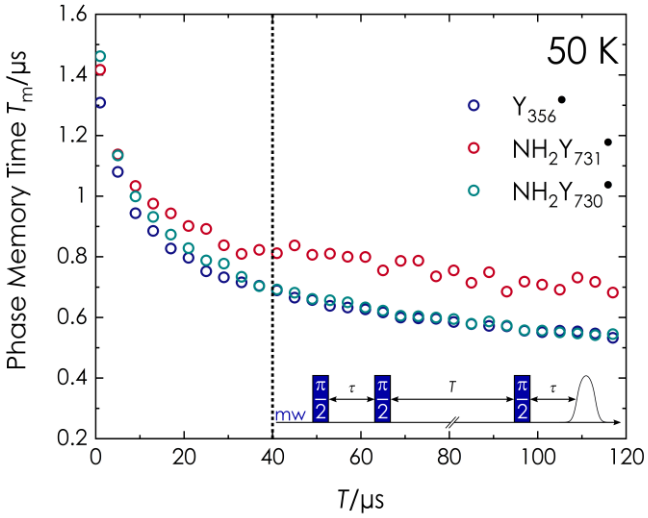

Figure S2: Phase memory times T_m as a function of the time T. Experimental values at 50 K for the three radicals $Y_{356}^{•}/Y_{730}F$-α (blue), $NH_2Y_{731}^{•}$ (red) and $NH_2Y_{730}^{•}$ (cyan). The time T = 40 μs, used in ENDOR experiments, is marked by a dotted line. Inset: microwave pulse sequence.

The experiments show that initially, T_m decreases almost exponentially with increasing pulse separation time T for all investigated radicals. At T = 40 μs, which was used for all ENDOR experiments, the phase memory time is approximately 700 - 800 ns for the three trapped radical intermediates.

SI1.4 ENDOR spectroscopy

ENDOR experiments at 94 and 263 GHz were recorded with the Mims[6] pulse sequence (π/2 - τ - π/2 – RF - π/2 – τ – echo) most sensitive to small hyperfine couplings. The microwave power at both instruments was reduced to produce π/2-pulses of 40 ns with an excitation bandwidth of 25 MHz/ 0.7 mT for increased orientation selectivity. The τ – value was set as explained in the following section (SI1.5). A 250 W RF-amplifier (250A250A, Amplifier research) was used to increase the RF pulse power. RF pulse length was optimized with a RF Rabi nutation experiment (see Fig. S3). At W-band frequency, 40 µs RF pulses with an excitation bandwidth of 25 kHz were used, while 75 µs pulses with an excitation bandwidth of 13 kHz were used at 263 GHz. The difference in experimental setup at 3.4 T vs 9.4 T is caused by the different ENDOR resonator design and efficiency as well as varying output powers of the amplifier at the different frequencies.

All ENDOR experiments were recorded using stochastic RF acquisition with 30 shots per point (SPP).[7-9] Comparison of experiments with 1 SPP vs 30 SPP showed negligible saturation effects (data not shown), while a significant shortening of measurement time was observed for the latter method. This is caused by the reprogramming time of the spectrometer upon change of the RF frequency (i.e. between each *x*-axis data point), which is around 30 ms and does not occur between shots at the same frequency. ENDOR experiments were recorded at 50 K at W-Band and at 20 K at 263 GHz. The temperature was chosen to achieve the best compromise between high signal intensity and short relaxation time for quick experimental shot repetition.

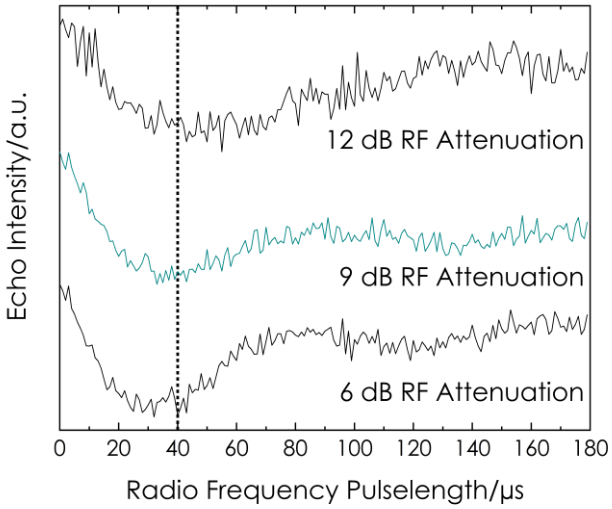

Figure S3: Rabi nutation experiments of ^{17}O nuclei at 6, 9 and 12 dB radio frequency attenuation at 3.4 T (W-band). The nutation with the experimentally used attenuation is colored in cyan and the used radio frequency pulse length is marked by a dotted line. Experimental parameters: Temperature = 50 K, pulse sequence: π/2 - τ - π/2 – RF - π/2 - τ - echo, π/2 = 10 ns, τ = 390 ns, RF = 1->180 µs, 30 shot/point, 5 ms SRT.

SI1.5 Choice of τ – value for Mims ENDOR

The sensitivity S of the Mims ENDOR experiment is described by the product of the ENDOR efficiency (F_{ENDOR}) and the echo intensity I_{echo}:

$$S = F_{\mathrm{ENDOR}} \cdot I_{\mathrm{echo}} \quad (3)$$

The Mims ENDOR efficiency for a nuclear spin ½ system can be described by a periodic function, depending on the effective hyperfine coupling A_{eff} :[10]

$$F_{\mathrm{ENDOR}} = \frac{1}{2}\sin^2\left(2\pi\frac{A_{\mathrm{eff}}}{2}\tau\right) \quad (4)$$

This formula breaks down for nuclear spins $I > ½$, if the quadrupole coupling is on the order of the hyperfine coupling.[11] The approach was adapted for $I = 1$ nuclei by calculating A_{eff} from a combination of hyperfine and quadrupole coupling by Hoffman and coworkers. A similar treatment to $I = 5/2$ nuclei was however deemed unfeasible.[11] Therefore, the blind spots in a ^{17}O Mims ENDOR spectrum have to be treated within the density matrix formalism and an explicit calculation of the coherence transfer pathway of the Mims sequence for each individual spin system of interest. This can be achieved with the easyspin routine *saffron*[12] (vide infra). The second term on the right side of equation 3 nevertheless is true and has to be considered, since I_{echo} decays exponentially as a function of the phase memory time T_m (see Eq. 2). The choice of the optimal τ–value therefore depends on T_m (here ~ 0.7 µs, see Fig. S2) and the expected coupling parameters (A and P).

Fig. S4 shows the simulated ENDOR spectrum of $Y_{356}^{\bullet}$ with blind spots (color, Mims ENDOR simulation) and with pure tensor simulation (black). The ENDOR spectra are scaled by the phase memory time. The simulations show that no periodic blind spots are clearly visible in the simulated spectra. The shape of the central nuclear transitions depends slightly on τ but the main difference is the change in overall signal intensity. The overall maximum ENDOR signal can be achieved with values between 400 and 600 ns. In this study, we chose a τ-value of 390 ns to give the best compromise between ENDOR sensitivity and echo intensity, since the latter also influences the overall signal-to-noise of the spectra.

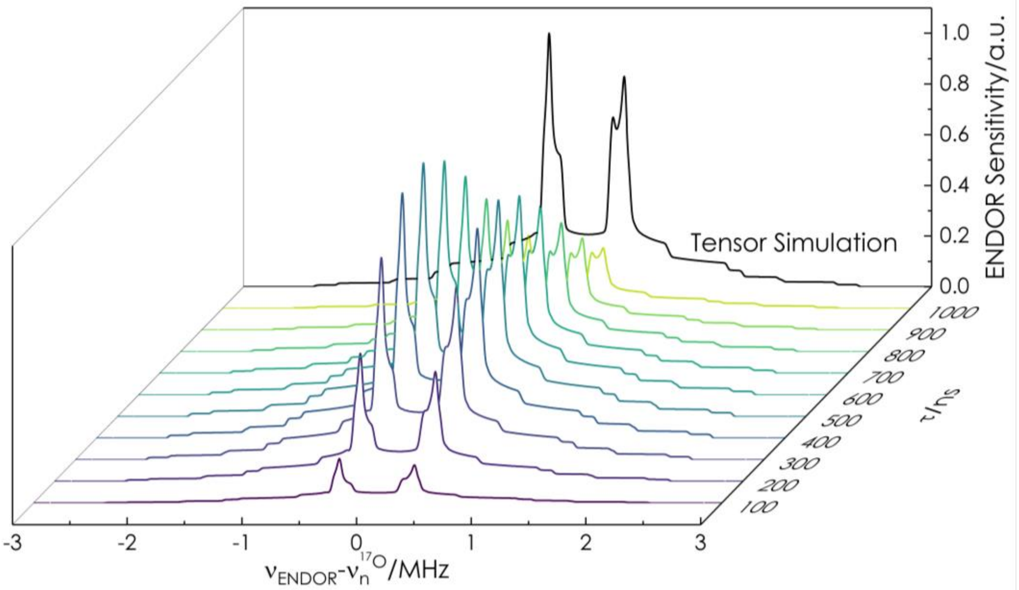

Figure S4: Comparison of ENDOR spectra of $Y_{356}^{\bullet}$ simulated with different τ-values (color) and with pure tensor simulation (black). Simulations are scaled by the experimentally determined phase memory time T_m = 0.7 µs.

SI1.6 Simulation of ENDOR spectra

All ENDOR spectra were simulated using the EasySpin software package.[13, 12] The simulated spin system was based on the literature *g*-values of the radicals $Y_{356}^{\bullet}$[5], $NH_2Y_{731}^{\bullet}$ and $NH_2Y_{730}^{\bullet}$[3] as well as the nuclei with the largest hyperfine coupling constants (Table S2). In case of $Y_{356}^{\bullet}$, this was only the β-methylene proton, while the amino nitrogen was included for $NH_2Y_{731}^{\bullet}$ and $NH_2Y_{730}^{\bullet}$. Additional couplings were neglected, since they significantly prolong calculation times while their contribution to the orientation selection and therefore the simulated ^{17}O ENDOR spectra was found to be negligible. ^{17}O ENDOR spectra were calculated with the *saffron* routine employing full tensor diagonalization (See S4). An excitation bandwidth of 25 MHz was used to select the orientations. A uniform ENDOR linewidth of 60 kHz was used for all simulations. All simulated ENDOR spectra were normalized to unity for comparison with equally treated experimental spectra. The quadrupole coupling size calculated by DFT was generally too large, evident from simulation of the ENDOR spectra. The deviation can in part be explained by the absence of other hydrogen bonding partners to the water molecule in the small DFT models, which are known to reduce the quadrupole coupling constant.[14] Therefore, the literature known coupling constants for pure water in ice: ***P***={-0.02 -0.32 0.34} MHz[14] was chosen and found to be in good agreement with the data of this work.

Table S2: Spin system parameters for EPR/ENDOR simulations[3, 5]

Radical	g_x	g_y	g_z	Nuclei	A_x	A_y	A_z
$Y_{356}^{\bullet}$	2.0062	2.0045	2.0022	H_β	61	52	57
$NH_2Y_{731}^{\bullet}$	2.0051	2.0040	2.0022	H_β<br>N_{amino}	23<br>2	22<br>3	22<br>31
$NH_2Y_{730}^{\bullet}$	2.0054	2.0042	2.0022	H_β<br>N_{amino}	31<br>2	29<br>3	28<br>31

SI1.7 DFT calculations

DFT models were calculated using the Orca 4.0.1.2 software package.[15] Geometry optimization was performed using the BP86 functional[16-17] in combination with the Ahlrichs' def2-TZVP basis set of triple-ζ quality[18-19] for all atoms and the RIJCOSX approximation(def 2/J auxiliary basis set)[20]. Grimmes dispersion correction (d3bj)[21-22] was applied on top of the SCF calculations. Single point energies and EPR parameters were calculated from the geometry optimized structures employing the B3LYP[17, 23-24] functional in conjunction with the EPR-II basis set for all nuclei.[25] The abovementioned RIJCOSX approximation and dispersion correction were also used. The protein environment was approximated by a conductor–like polarization model (CPCM) with polarity $\varepsilon = 24$.

The geometry of the small models of a tyrosyl and amino-tyrosyl radical model was initially optimized without a water molecule and only restricted to the experimentally known β-H dihedral angles (C_2–C_1–C_β-$H_{\beta 1}$) of 70° and -120° for $Y_{356}^\bullet$ and $NH_2Y_{731}^\bullet$, respectively.[3, 5] A water molecule was added and its geometry was optimized while the C_3-C_4-$O_{Tyr} \cdots H_{H2O}$ dihedral angle θ and the coordinates of all the radical atoms were fixed. In case of the amino tyrosyl radical, the amino protons were not fixed, since they are potential hydrogen bond partners for the water molecule. 36 individual models were calculated with θ values in increments of 10° from 0° to 350°. The $O_{Tyr} \cdots H_{H2O}$ distance r was not fixed in the models.

SI2 Radical yield determination

Due to the half-site reactivity of *E.coli* RNR[26-27], the EPR spectra of hand quenched samples contain the contribution of two radicals. One signal is the trapped radical in the RT pathway of one α/β pair of the active α2β2 complex: Y_{356}•/Y_{730}F-α (Fig. S5A), NH_2Y_{731}• (Fig. S5B) or NH_2Y_{730}• (Fig. S5C). The second signal is Y_{122}• or F_3Y_{122}• in the unreacted α/β pair. The two radical species have very different relaxation times due to their different environments. The signal associated with the radical at residue 122 relaxes very fast due their proximity to the di-iron center, making it fully visible only at very low temperatures.[28] Therefore, echo-detected EPR spectra of the samples were recorded at 10 K (Fig. S5, red lines) and the EPR spectrum of the respective tyrosyl radical at residue 122 (Fig. S5, blue lines) was subtracted.[29] The relative amount of radical trapped was then determined from the integral of the full EPR signal versus the integral of the subtracted spectrum (Fig. S5, cyan lines). The resulting radical yields are displayed in Fig. S5 and Table S1.

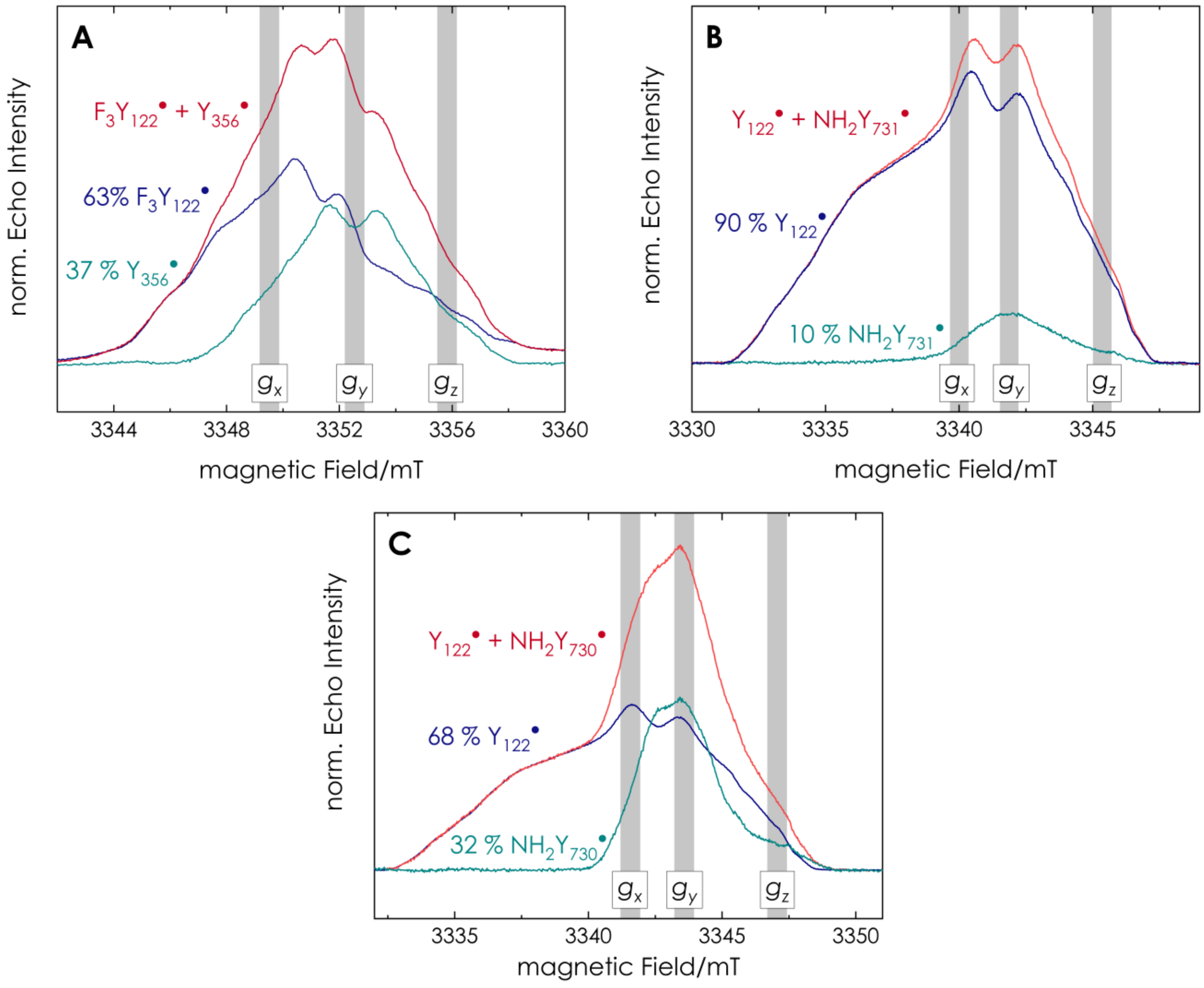

Figure S5: Echo-detected EPR spectra of reaction mixtures (red) with the radicals Y_{356}•/Y_{730}F-α2 (A), NH_2Y_{731}• (**B**) and NH_2Y_{730}• (**C**), reference spectra of the respective resting β_2 (blue) and subtraction of the two spectra (cyan). Relative amounts of radical determined by integration and given in the figure. Canonical *g*-Tensor orientations ($B_0 \| g_x$, $B_0 \| g_y$ and $B_0 \| g_z$) at which the orientation selective ENDOR spectra were recorded are marked by gray areas. Experimental parameters: Temperature = 10 K, Pulse Sequence: π/2 - τ - π - τ - echo, π/2 = 10 ns, τ = 290 ns, 5 shot/point, 100 ms SRT.

SI3 Mims ENDOR spectra of $Y_{122}^{\bullet}$ and $F_3Y_{122}^{\bullet}$

The ^{17}O ENDOR experiments in this study were performed on radical mixtures that contain more than 50% unreacted tyrosyl radical $Y_{122}^{\bullet}$ or $F_3Y_{122}^{\bullet}$ at the diiron cofactor. (See Figure S5 and Table S1). To exclude ^{17}O hf contributions of this radical, Mims ENDOR experiments of $Y_{122}^{\bullet}$ and $F_3Y_{122}^{\bullet}$ in the spectral region investigated in all other ENDOR experiments (19.3 ± 3 MHz) were recorded. These ENDOR experiments were performed at 10 K due to the fast relaxation properties of the two radicals caused by the adjacent di-iron cluster (see SI2). Experiments were performed at the maximum of the EPR signal, i.e. $B_0 \| g_y$ and with full microwave power to increase the ENDOR signal. The ENDOR spectra show a small amount of a mostly featureless signal in a range of ±0.2 MHz around the ^{17}O Larmor frequency. This contribution is smaller at temperatures higher than 10 K due to the fast relaxation properties which cause signal loss. The results confirm that the distinct coupling features detected in the ^{17}O ENDOR experiments of the pathway radical intermediates originate from water molecules at the intermediates themselves and not from the tyrosyl radicals associated with the di-iron cofactor.

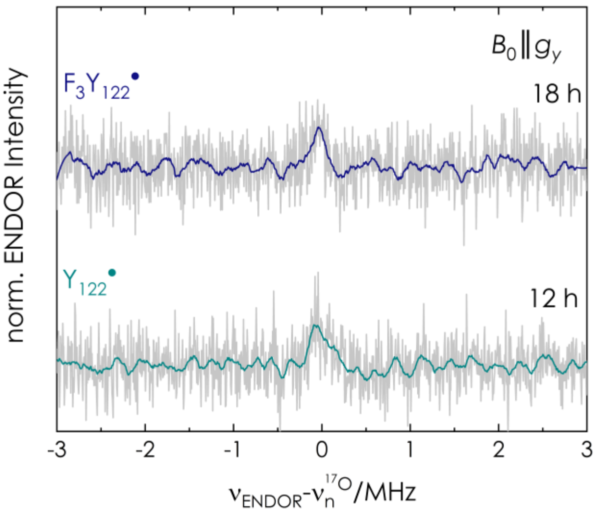

Figure S6: Mims ENDOR spectra of $Y_{122}^{\bullet}$ (cyan) and $F_3Y_{122}^{\bullet}$ (blue) in a region of ±3 MHz around the ^{17}O Larmor frequency recorded at the maximum EPR intensity, i.e. $B_0 \| g_y$. Experimental parameters: Temperature = 10 K, Pulse Sequence: $\pi/2$ - τ - $\pi/2$ - RF - $\pi/2$ - τ - echo, $\pi/2$ = 8 ns, τ = 390 ns, RF = 40 µs, 5 shot/point, random rf acq., 30 ms SRT, 6 kHz RF sweep interval. Acquisition time of the spectra is written in the figure.

SI4 ENDOR spectra of I=5/2 nuclei

Nuclear spins $I > 1/2$ such as ^{17}O ($I = 5/2$) exhibit quadrupole coupling due to the interaction between the electric field gradient and the non-uniform charge distribution within the nucleus. The interaction is described by the traceless quadrupole coupling tensor $\boldsymbol{P}$ in the nuclear quadrupole Hamiltonian and causes an energy shift of the nuclear spin states in addition to the hyperfine interaction.[30]

$$\boldsymbol{P} = \frac{e^2 qQ}{4I(2I-1)} \cdot \begin{pmatrix} -(1-\eta) & & \\ & -(1+\eta) & \\ & & 2 \end{pmatrix} \quad (5)$$

The spin Hamiltonian for a coupled electron S = 1/2 to a nuclear spin I = 5/2 is described by the spin Hamiltonian:[30]

$$\widehat{\boldsymbol{H}}\left(S = \frac{1}{2}, I = \frac{5}{2}\right) = \mu_B \boldsymbol{B}\boldsymbol{g}\widehat{\boldsymbol{S}} - \mu_N g_n \boldsymbol{B}\widehat{\boldsymbol{I}} + \widehat{\boldsymbol{S}}\boldsymbol{A}\widehat{\boldsymbol{I}} + \widehat{\boldsymbol{I}}\boldsymbol{P}\widehat{\boldsymbol{I}} \quad (6)$$

If the hyperfine and quadrupole interactions are small as compared to the nuclear Zeeman interaction, they can usually be described within the high-field approximation, which significantly simplifies the spin Hamiltonian and makes simulation of the system fast. In the high-field approximation, only the zz-components of the coupling tensors are taken into account, assuming that $\widehat{\boldsymbol{S}}_z$ and $\widehat{\boldsymbol{I}}_z$ are dominating:

$$\widehat{\boldsymbol{H}}\left(S = \frac{1}{2}, I = \frac{5}{2}\right) = \mu_B \boldsymbol{B}\boldsymbol{g}\widehat{\boldsymbol{S}}_z - \mu_N g_n \boldsymbol{B}\widehat{\boldsymbol{I}}_z + \widehat{\boldsymbol{S}}_z A_{zz}\widehat{\boldsymbol{I}}_z + \widehat{\boldsymbol{I}}_z P_{zz}\widehat{\boldsymbol{I}}_z \quad (7)$$

If the high field approximation is justified, simulation of the ENDOR spectra with high field approximation and full matrix diagonalization should yield identical spectra. Figure S7 shows the simulated ENDOR spectra for a series of spin systems (Table S3) with coupling values similar to the ones in this work simulated with the two methods at a magnetic field of 3.35 T, which corresponds to the one used in the majority of experiments in this work. We simulated the individual nuclear transitions (color) and the full spectrum (black) to better illustrate the effect of nuclear quadrupole coupling on the spectral line shape.

Table S3: Hyperfine and quadrupole coupling parameters for Fig. S7. All values in MHz.

Simulation	a_{iso}	T_x	T_y	T_z	P_x	P_y	P_z
A	0	-0.2	0.1	0.1	-0.001	-0.001	0.002
B	0.6	0	0	0	-0.001	-0.001	0.002
C	0.6	-0.2	0.1	0.1	-0.001	-0.001	0.002
D	0	-0.2	0.1	0.1	-0.17	-0.17	0.34
E	0.6	0	0	0	-0.17	-0.17	0.34
F	0.6	-0.2	0.1	0.1	-0.17	-0.17	0.34
G	0	-0.2	0.1	0.1	-0.02	-0.32	0.34
H	0.6	0	0	0	-0.02	-0.32	0.34
I	0.6	-0.2	0.1	0.1	-0.02	-0.32	0.34

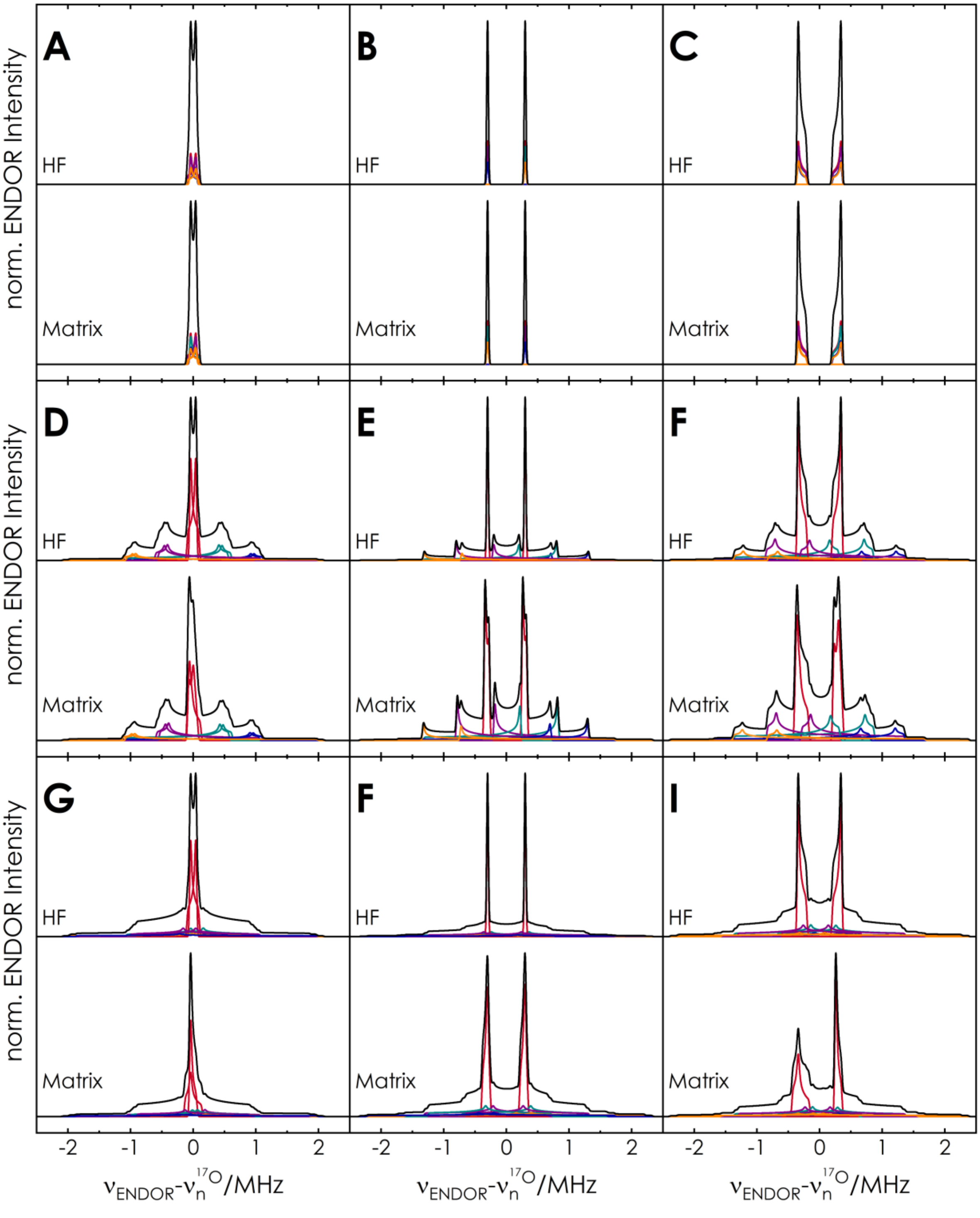

Figure S7: Simulated ENDOR spectra (black) and individual nuclear transitions (color) for a coupled spin system S=1/2 I=5/2 calculated in high field approximation with 1st order perturbation theory (HF) and with full tensor diagonalization (Matrix). Coupling parameters are reported in Table S3.

The first three spin systems (A, B and C) have very small quadrupole coupling constants and purely dipolar (A), purely isotropic (B) or combined (C) hyperfine coupling tensors. The nuclear transitions within the two electron spin manifolds have almost identical frequencies and only vary in intensity due to different transition probabilities. The simulated spectra for purely dipolar hf coupling show in both cases a small doublet structure close to the larmor frequency (A). In case of isotropic hf coupling (B) the signals are very sharp and only broadened by the ENDOR linewidth. In case of the rhombic hf tensor (C) the signals are additionally broadened by the dipolar hf coupling. For these systems, both methods yield the same result, justifying the high field approximation.

The second set of spin systems (D, E and F) have axial quadrupole tensors in the size observed experimentally and either dipolar (D), isotropic (E) or combined (F) hyperfine coupling tensors. Even though this quadrupole tensor shape is not expected in the systems of this study, the simulations are shown here to better illustrate the impact of quadrupole coupling on the ENDOR spectra. The individual nuclear transitions in the two electron spin manifolds are no longer energetically equivalent and the simulated ENDOR spectra contain sharp central signals (red) split by the hyperfine coupling, which correspond to the m_S= -1/2 to m_S=+1/2 nuclear transition as well as broad signals corresponding to the other nuclear transitions. The central transitions are not affected by quadrupole coupling in the high field approximation, so they have the same line shape as in A, B and C. The simulations performed with matrix diagonalization show that the central transitions are affected by the quadrupole coupling and become broadened and in case of a hf coupling tensor with dipolar coupling contribution (D and F) also asymmetric around the ^{17}O Larmor frequency. For a small, purely dipolar tensor (D) the small doublet structure is lost and the signal at the Larmor frequency becomes a single asymmetric peak.

The third set of spin systems has rhombic quadrupole coupling tensors (G, H and I), equivalent to the systems we investigated in this work. The quadrupole transitions become much broader and therefore less intense due to their rhombic tensor shape. In case of small dipolar coupling (G) the effect observed in the previous section is amplified and all hf coupling information is lost in the spectrum within a single central peak. For the case of isotropic hf coupling (H) the broadening effect on the central transition is limited while a significant broadening and asymmetry is observed for rhombic hf and quadrupole tensor (I).

We conclude, that the high-field approximation fails for the systems investigated in this work and full matrix diagonalization has to be used for all spectral simulations. We also conclude that the asymmetry of the ENDOR spectra is caused by the size and shape of the hyperfine and quadrupole tensors and therefore has to be considered during simulation.

SI5 Orientation selective Mims ^{17}O ENDOR spectra of the radical intermediates

Mims ENDOR spectra of the three radical intermediates $Y_{356}^{\bullet}$ (Y_{730}F-α)(A), $NH_2Y_{731}^{\bullet}$ (B) and $NH_2Y_{730}^{\bullet}$ (C) were recorded at the field positions corresponding to the three canonical *g*-tensor orientations marked in Fig. S5: $B_0 \| g_x$ (blue), $B_0 \| g_y$ (red) and $B_0 \| g_z$ (cyan). Baseline corrected (1st order polynomial) spectra are shown in light gray and 4th order Savitzky-Golay filtered spectra (20 pt window) are shown in color. The difference in signal-to-noise in the spectra is a consequence of several factors: a) radical yield of the sample with the lowest overall S/N for $NH_2Y_{731}^{\bullet}$, b) different EPR signal intensity at the field positions with $B_0 \| g_y$ giving the best S/N since it is the maximum of the EPR spectrum, c) different ENDOR sensitivity at the specific *g*-tensor orientations with $B_0 \| g_z$ having more ENDOR sensitivity than $B_0 \| g_x$ for the $Y_{356}^{\bullet}$ radical, whiles this is reversed for the amino-tyrosyl radicals. This is caused by the smaller difference of g_x and g_y in the amino-tyrosyl radicals, resulting in the excitation of more orientations and the larger similarity of the g_x and g_y spectra. Orientation selective ENDOR simulations with the spin system parameters specified under SI 1.6 and the ^{17}O coupling parameters from Table S4 are shown in black.

Table S4: Simulated and DFT calculated 17O hyperfine and quadrupole coupling parameters

Radical	A_x	A_y	A_z	α	β	γ	P_x	P_y	P_z	α	β	γ
$Y_{356}^{\bullet}$	0.43<br>0.19	0.66<br>0.59	0.70<br>0.65	49	168	-67	-0.02	-0.32	0.34	-39	87	-22
$NH_2Y_{731}^{\bullet}$	0.70	0.84	0.89	84	109	-68	-0.02	-0.32	0.34	50	82	-27
$NH_2Y_{730}^{\bullet}$	0.65<br>0.24	0.80<br>0.6	0.89<br>0.6	84	109	-68	-0.02	-0.32	0.34	50	82	-27

DFT values are shown in blue. All coupling constants given in MHz. Euler angles given in degrees and defined from the *A* and *P* to the *g* tensor based on the *y* convention. *A* and *P* tensors are chosen so that $|A_x/P_x| < |A_y/P_y| < |A_z/P_z|$.

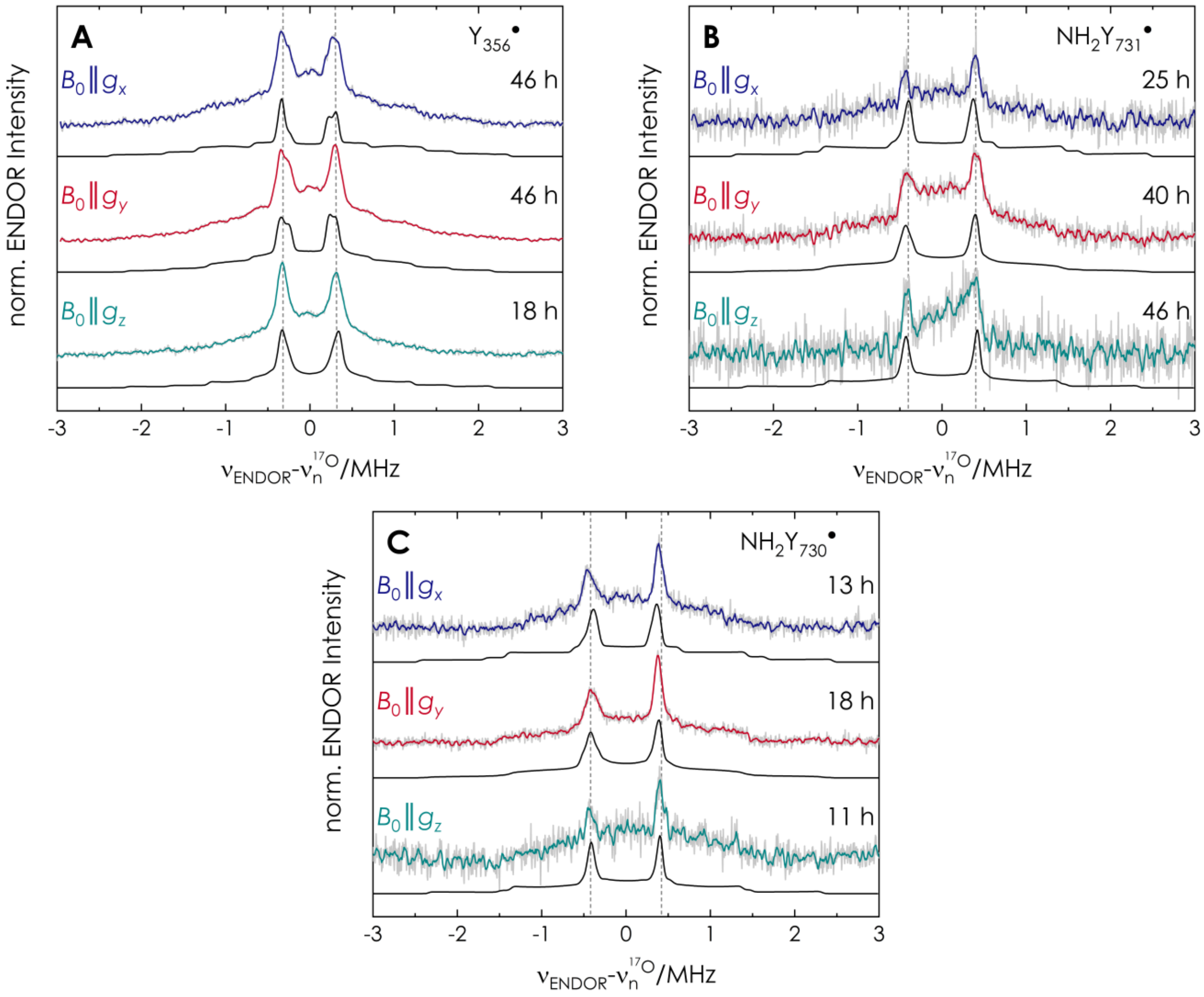

Figure S8: Orientation selective Mims ENDOR spectra of Y_{356}• ($Y_{730}F$-α) (**A**), NH_2Y_{731}• (**B**) and NH_2Y_{730}• (**C**) recorded at field positions corresponding to the three g-tensor orientations $B_0 \| g_x$ (blue), $B_0 \| g_y$ (red) and $B_0 \| g_z$ (cyan) and their simulation (black). Experimental parameters: Temperature = 50 K, Pulse Sequence: π/2 - τ - π/2 - RF - π/2 - τ - echo, π/2 = 40 ns, τ = 390 ns, RF = 40 μs, 30 shot/point, random RF acquisition, 5 ms (Y_{356}•); 10ms ($NH_2Y_{731/730}$•) SRT, 6 kHz RF sweep interval. Acquisition time of the spectra is written in the figure. Simulation parameters given in section SI 1.6 and Table S4.

SI6 DFT models of an isolated tyrosyl radical with a coordinated water molecule

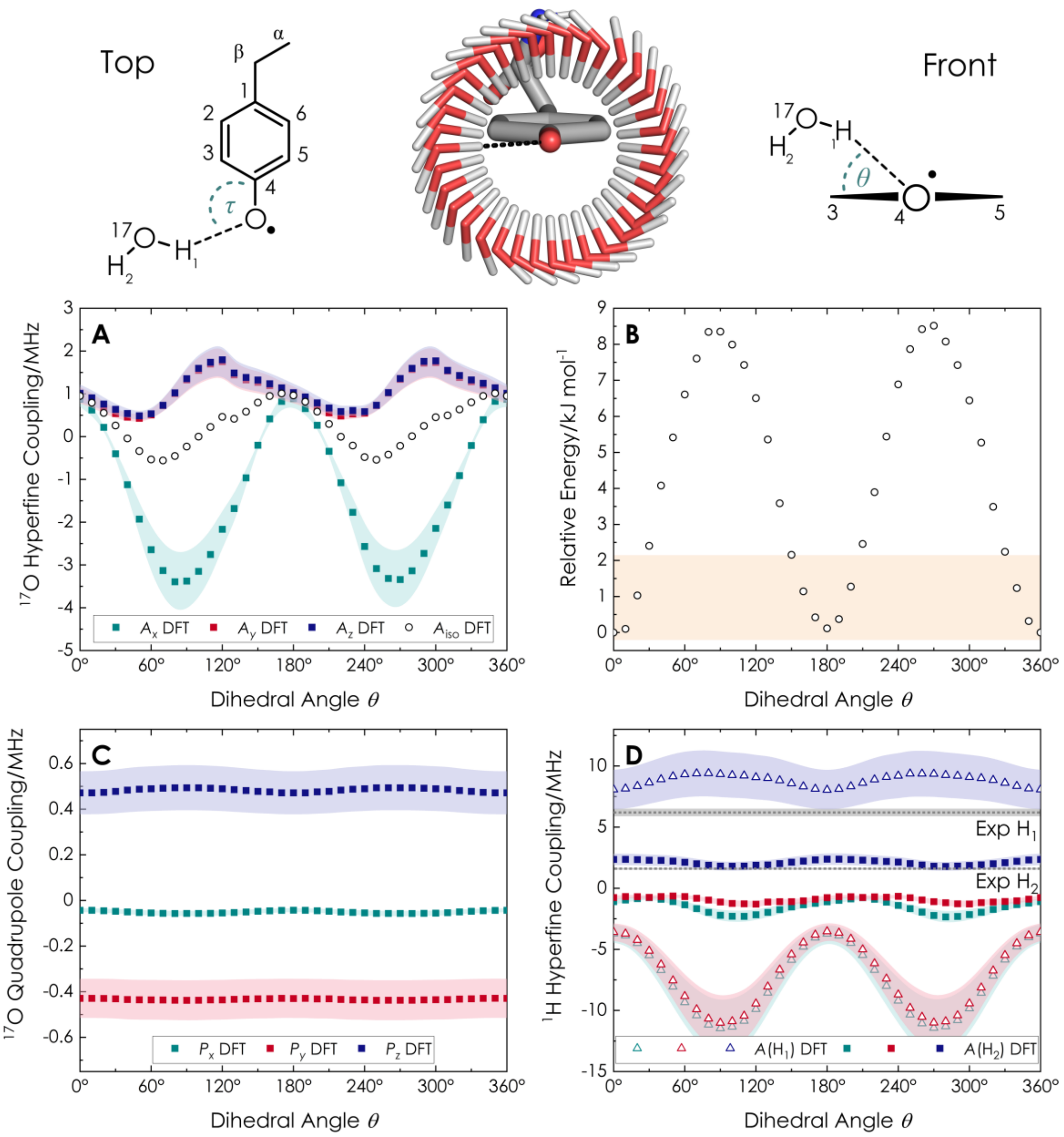

Figure S9: Angle scan for a tyrosyl radical with one water molecule. **Top:** Small (26 atoms) DFT models with one water molecule fixed at τ = 120° and dihedral angles C_3-C_4-O_{Tyr} ··· H_{H_2O} θ at DFT optimized distances of r (O_{Tyr} ··· H_{H_2O}) = 1.8 Å. 0° structure is marked by black dashed line. Calculation method described under SI 1.7. **A**: Calculated ^{17}O hf coupling tensor components A_x, A_y and A_z as well as isotropic coupling constant A_{iso}. Almost isotopic coupling in the experimental range ≲ 1 MHz is found for θ in the range ≲ ± 30° and 150° ≲ θ ≲ 240°. **B**: Relative energy of the calculated structures, θ = 0° structure set as the zero-point. Orange area represents an interval of thermal energy (kT) at 298 K. Coordination with θ = 0° and 180° results in equivalent energetic minima. **C**: Calculated ^{17}O quadrupole coupling tensor components P_x, P_y and P_z. The values change only slightly as a function of θ. **D**: Calculated ^{1}H hf coupling tensors components A_x (cyan), A_y (red) and A_z (blue) for H_1 (open triangles) and H_2 (full squares). Experimental values A from previous study[5] marked by dashed lines with 5 % confidence interval marked by grey areas. Couplings close to the experimental values are observed for θ = 0° and 180°. 20 % confidence interval of DFT calculated coupling constants are marked by colored areas.

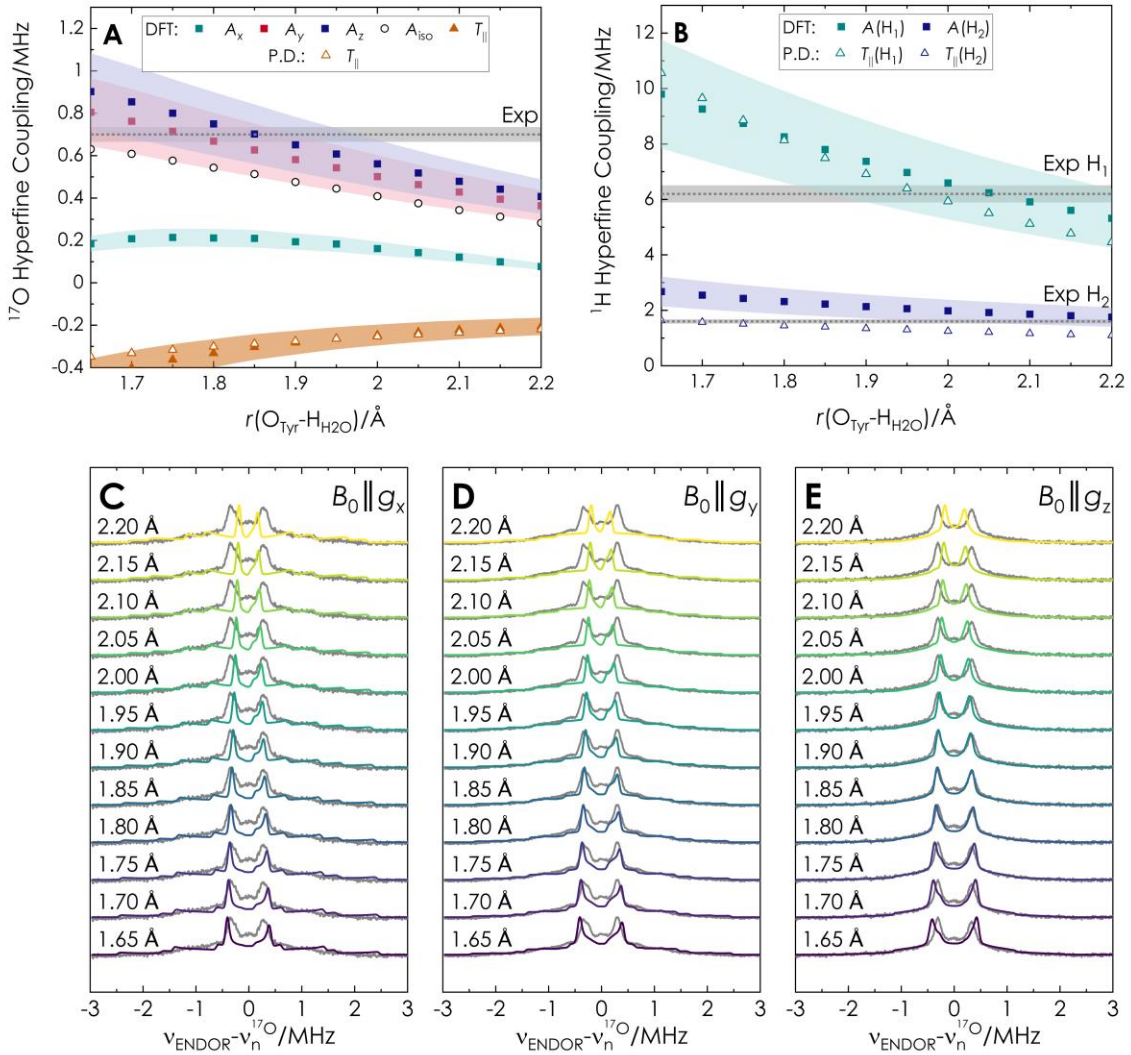

Figure S10: Distance scan for a tyrosyl radical with one water coordinated at $\theta = 20°$. **A**: DFT calculated ^{17}O hyperfine coupling tensor components and point-dipole (P.D.) approximated $T_{||}$ components using a spin density = 0.3 on the oxygen atom of Y$^{\bullet}$. **B**: DFT calculated ^{1}H hf coupling tensor components and point-dipole (P.D.) approximated $T_{||}$ components with spin density $\rho = 0.3$. Experimental values are shown as gray dashed lines with a 5 % confidence interval marked as grey area. 20 % confidence interval of DFT calculated coupling constants are marked by colored areas. Orientation selective ^{17}O Mims ENDOR simulations at **C**: $B_0 \| g_x$, **D** $B_0 \| g_y$. **E:** $B_0 \| g_z$ for the calculated coupling tensors as a function of the r(OH) distance.

Figure S10 displays the distance dependence of the ^{17}O (A) and ^{1}H hf (B) couplings calculated by the DFT distance scan as well as by a simple point-dipole model. The dipolar coupling contribution from the point-dipole (P.D.) model was estimated as:

$$T_{||}(\text{P.D.}) = 2 \cdot T = 2 \cdot \frac{\mu_0}{4\pi h} g_e \mu_B g_n \mu_N \frac{1}{r^3} \cdot \rho \quad (8)$$

where r is the inter spin distance and ρ the spin density. Since the water molecule is coordinated almost in plane, we assumed an interaction only with the spin density ρ on the oxygen atom, which

was estimated from the DFT Loewdin spin population[31] analysis calculation to be 0.3 for the tyrosyl radical. Nuclear g values used were: $g_n(^{17}O)$ = -0.7575 and $g_n(^{1}H)$ = 5.5857.[32]

$$T_{||}(^{17}\mathrm{O}) = -2 \cdot 10.722 \frac{\text{Å}}{r_{\mathrm{OO}}^3} \cdot 0.3 \quad \text{and} \quad T_{||}(^{1}\mathrm{H}) = 2 \cdot 79.064 \frac{\text{Å}}{r_{\mathrm{OH}}^3} \cdot 0.3 \quad (9)$$

We note that the point-dipole model agrees well with the dipolar contribution of ^{17}O computed by DFT (Figure S10A). For the ^{1}H coupling, the experimental values A correspond to $T_{||}$, as the tensors are almost purely dipolar. For the two ^{1}H tensors, the DFT computed values slightly exceed the point-dipolar approximation. The experimental values are found right between these two (Figure S10B).

Overall, the distance analysis shows that a H bond distance of 1.85 ± 0.05 Å leads to the best agreement of the ^{17}O couplings detected in this work, while a distance of ~ 1.95 ± 0.05Å leads to the best agreement with the ^{1}H couplings detected in Nick *et al*[5]. Taken this information together, we conclude that the H bond distance is 1.9 ± 0.05 Å.

Figure S10C,D,E illustrates that simulation of the ^{17}O ENDOR spectra with hf couplings predicted for a distance of 1.9 Å shows peaks and line shapes well compatible with the data.

SI7 DFT models of an isolated amino tyrosyl radical with a coordinated water molecule

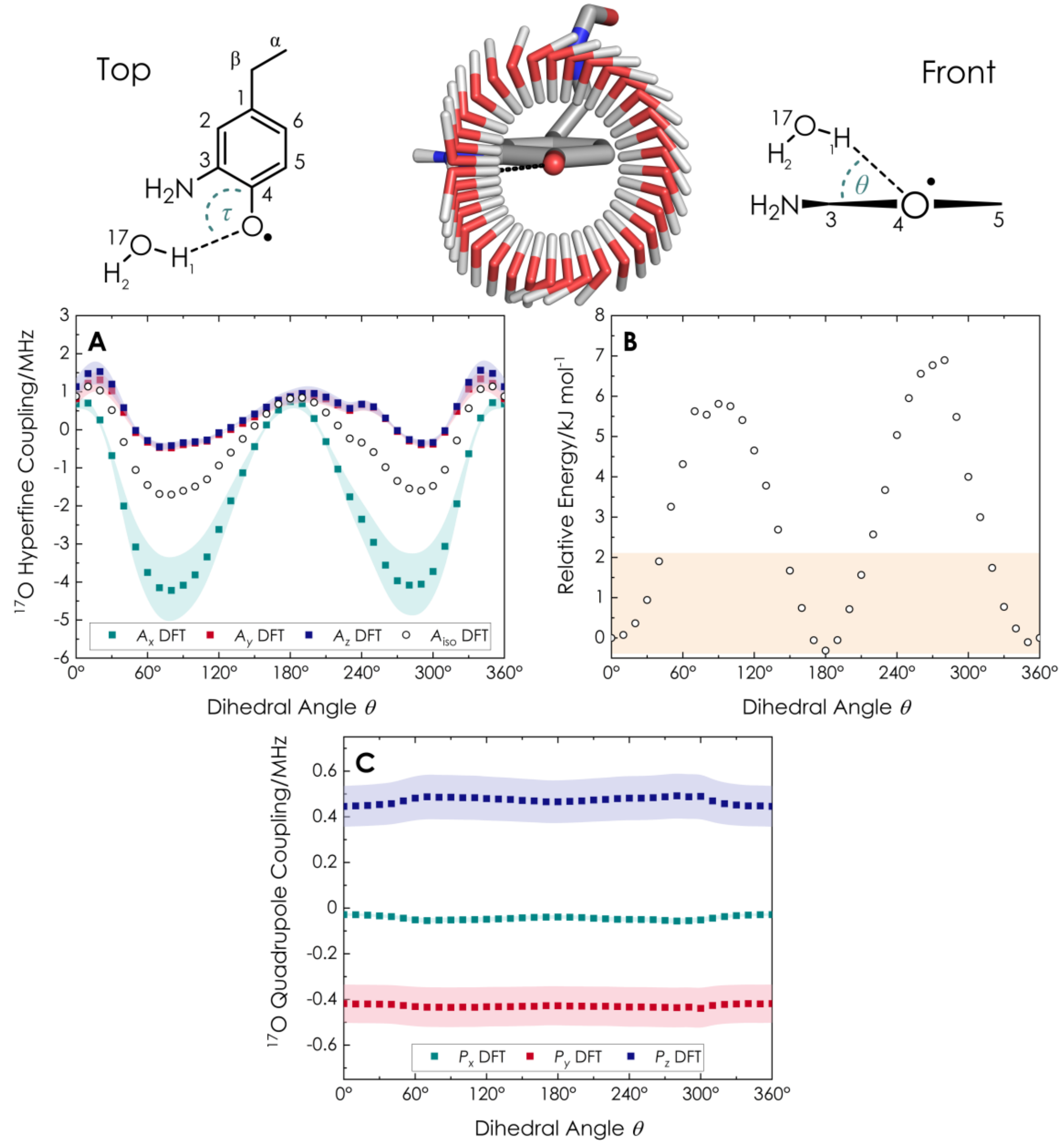

Figure S11: Angle scan for an amino-tyrosyl radical with one water molecule. **Top:** Small (27 atoms) DFT models with one water molecule fixed at dihedral angles C_3-C_4-O_{Tyr} ⋯ H_{H_2O} θ at DFT optimized distances of r (O_{Tyr} ⋯ H_{H_2O}) = 1.8 Å. τ was not fixed to account for the influence of the amino-group. 0° structure is marked by a black dashed line. Calculation method described under SI 1.7. **A**: Calculated ^{17}O hf coupling tensor components A_x, A_y and A_z as well as isotropic coupling constant A_{iso}. Almost isotopic coupling in the experimental range $\lesssim$ 1 MHz is found for θ in the range $\lesssim \pm$ 10° and 150° $\lesssim \theta \lesssim$ 240°. **B**: Relative energy of the calculated structures, θ = 0° structure set as the zero-point. Orange area represents an interval of thermal energy (kT) at 298 K. Coordination with θ = 180°, i.e. opposite to the NH_2-group, results in the energetic minimum. **C**: Calculated ^{17}O quadrupole coupling tensor components P_x, P_y and P_z. The values change only slightly as a function of θ. 20 % confidence interval of DFT calculated coupling constants are marked by colored areas.

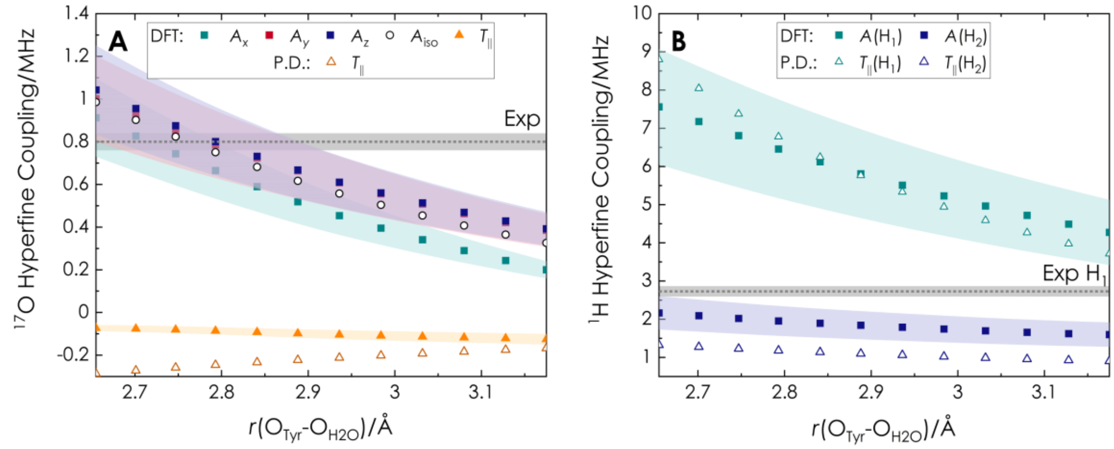

Figure S12: Distance scan for an amino tyrosyl radical with one water coordinated at $\theta = 180°$. **A:** Calculated ^{17}O hyperfine coupling tensor components and point-dipole (P.D.) approximated $T_{||}$ components (Eq. 7) with a calculated spin density of 0.25 on the oxygen atom of $NH_2Y^{\bullet}$. **B:** DFT calculated ^{1}H hf coupling tensor components and point-dipole (P.D.) approximated $T_{||}$ components with a calculated spin density $\rho = 0.25$. Experimental values are shown as gray dashed lines with a 5 % confidence interval marked as grey area. 20 % confidence interval of DFT calculated coupling constants are marked by colored areas.

Overall, the angle sweep for the amino-tyrosyl radical shows that coordination with θ=180°, i.e. in ring plane opposite to the NH_2-group results in the energetic minimum and ^{17}O hyperfine couplings compatible with the experimental data. A distance sweep for this coordination angle shows, that the experimental ^{17}O couplings of $NH_2Y_{730}^{\bullet}$ and $NH_2Y_{731}^{\bullet}$ would be most compatible with $r(O_{Tyr} \cdots O_{H2O})$ = 2.8 Å. (Fig. 12A) This distance is however not compatible with the experimentally observed ^{1}H hf couplings for $NH_2Y_{730}^{\bullet}$ (Fig. 12B) (and also with those of $NH_2Y_{731}^{\bullet}$ since they are even smaller. See Table 1). We conclude that the small model cannot describe the amino tyrosyl radicals sufficiently due to the absence of surrounding second sphere residues.

SI8 ^{17}O ENDOR of different mutants to generate $Y_{356}^{\bullet}$

The intermediate $Y_{356}^{\bullet}$ is accessible from different biochemical constructs which share the mutation, β_2-F_3Y_{122}. An additional mutation of $Y_{730}F$ in the pathway results in the highest radical yield. The F_3Y_{122}-β_2/$Y_{730}F$-α_2 construct was thus investigated both at 94 and 263 GHz. The F_3Y_{122}/$E_{52}Q$-β_2/α_2 was also studied as this double mutation leads to a tight $\alpha_2\beta_2$ complex that is active[33] which gave rise to the first high-resolution structure of the holocomplex by cryo-EM.[34] Additionally, F_3Y_{122}-β_2/F_2Y_{731}-α_2 construct is planned for measurement of the distances between Y_{356} and the fluorines in F_2Y_{731}. Figure S13 shows 94 GHz Mims ENDOR experiments for four possible combinations of subunit pairs prepared under similar conditions. The ^{17}O ENDOR line shape and position of the peaks do not vary significantly. Minor differences in asymmetry can be explained by slight variations in field positions. The spectra generally show that a specific coordination of one water molecule exists and is conserved across the biochemical constructs.

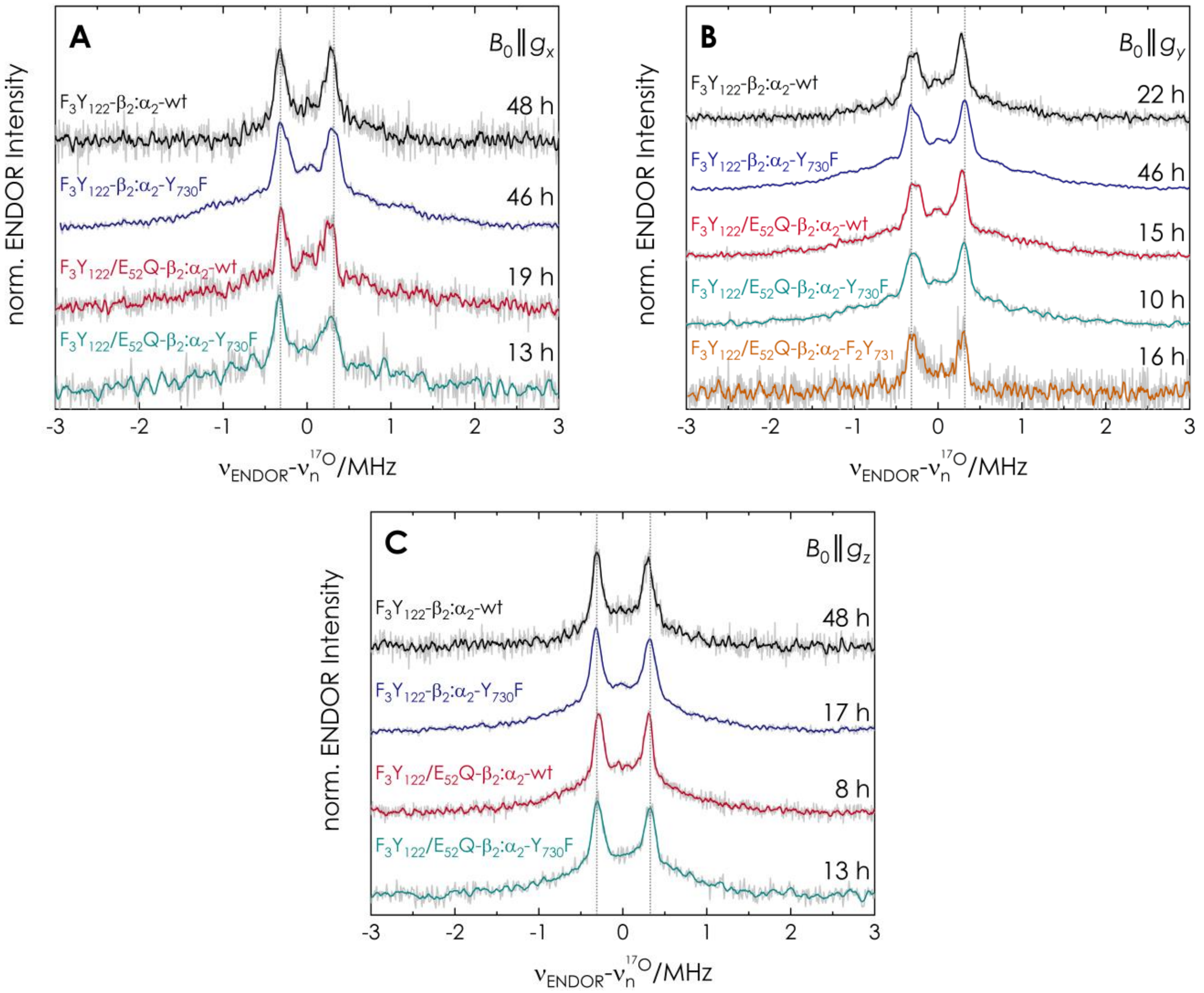

Figure S13: Orientation selective Mims ENDOR spectra of $Y_{356}^{\bullet}$ produced by different biochemical constructs. **A**: $B_0 \| g_x$, **B** $B_0 \| g_y$. **C:** $B_0 \| g_z$. Black: F_3Y_{122}-β_2 with wt-α_2. Blue: F_3Y_{122}-β_2 with $Y_{730}F$-α_2. Red: $F_3Y_{122}/E_{52}Q$-β_2 with wt-α_2. Cyan: $F_3Y_{122}/E_{52}Q$-β_2 with $Y_{730}F$-α_2. Orange: $F_3Y_{122}/E_{52}Q$-β_2 with F_2Y_{731}-α_2. S/N ratios across the spectra vary due to different radical yields at position 356 and different total acquisition times of the experiments. The radical yields are 25 % for F_3Y_{122}-β_2:α_2-wt, 40 % for F_3Y_{122}-β_2:α_2-$Y_{730}F$, 35 % $F_3Y_{122}/E_{52}Q$-β_2:α_2-wt, 30 % $F_3Y_{122}/E_{52}Q$-β_2:α_2-$Y_{730}F$ and 5 % $F_3Y_{122}/E_{52}Q$-β_2:α_2-F_2Y_{731}. Experimental parameters: Temperature = 50 K, Pulse Sequence: $\pi/2$ - τ - $\pi/2$ - RF - $\pi/2$ - τ - echo, $\pi/2$ = 40 ns, τ = 390 ns, RF = 40 µs, 30 shot/point, random rf acq., 5 ms SRT, 6 kHz RF sweep interval. Acquisition time of the spectra is written in the figure.

SI9 Previous large DFT model of $\alpha_2\beta_2$–NH_2Y_{730}•

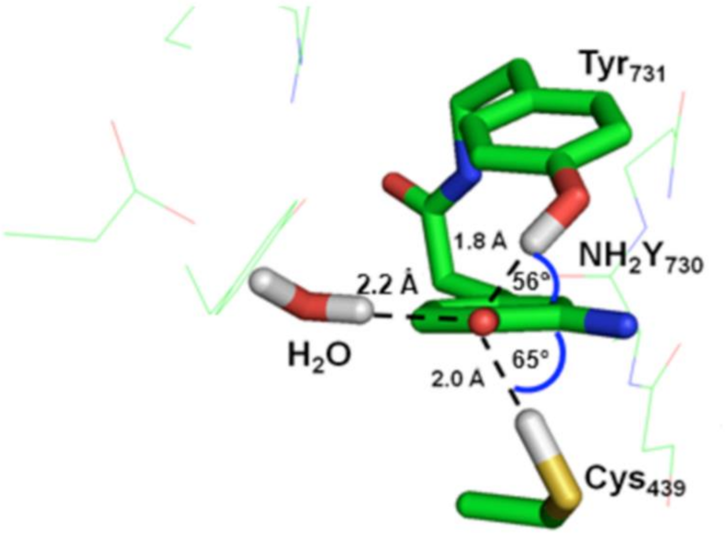

Figure S14: Combined EPR and DFT model (140 atoms) for the H-bond network around NH_2Y_{730}• in α_2 as reported in Argirevic *et al*, ref. [35]. Experimentally observed H bonds and are shown within the optimized DFT model. The water and its mechanistic significance was proposed in that study, but direct experimental evidence was missing. The present study assigns the H bond unambiguously to a water molecule. Hf coupling parameters of the water molecule are reported in the main text in Table 1 as DFT_{large}.

SI10 Previous large DFT models of $\alpha_2\beta_2$-$NH_2Y_{731}^{\bullet}$

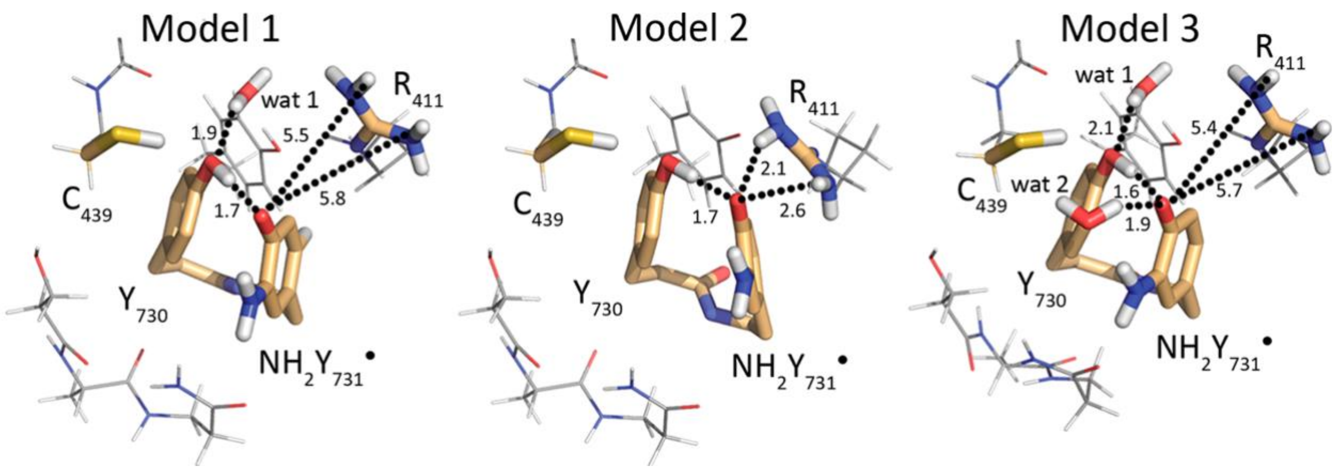

Figure S15: DFT optimized structures of $NH_2Y_{731}^{\bullet}$ as reported in Figure 5 of Nick *et al*, Ref. 3. Left: Model 1 includes only the water molecule wat1. Center: Model 2 has no water molecules. Model 3 contains a second water molecule wat2, which is observed in some X-ray structures. Residues in interaction distance are in gold. Distances are given in Å.

Table S5: Experimental and calculated hyperfine coupling parameters for wat2 in model 3

	A_x(^{17}O)	A_y(^{17}O)	A_z (^{17}O)	A (H$_1$)
Model 3	-0.05	0.14	-2.5	4.6
Experiment	0.7	0.84	0.9	≲ 2.5

REFERENCES

(1) Minnihan, E. C.; Young, D. D.; Schultz, P. G.; Stubbe, J., Incorporation of fluorotyrosines into ribonucleotide reductase using an evolved, polyspecific aminoacyl-tRNA synthetase. *J. Am. Chem. Soc.* **2011,** *133* (40), 15942-5.

(2) Lee, W.; Kasanmascheff, M.; Huynh, M.; Quartararo, A.; Costentin, C.; Bejenke, I.; Nocera, D. G.; Bennati, M.; Tommos, C.; Stubbe, J., Properties of Site-Specifically Incorporated 3-Aminotyrosine in Proteins To Study Redox-Active Tyrosines: Escherichia coli Ribonucleotide Reductase as a Paradigm. *Biochemistry* **2018,** *57* (24), 3402-3415.

(3) Nick, T. U.; Lee, W.; Kossmann, S.; Neese, F.; Stubbe, J.; Bennati, M., Hydrogen bond network between amino acid radical intermediates on the proton-coupled electron transfer pathway of E. coli alpha2 ribonucleotide reductase. *J. Am. Chem. Soc.* **2015,** *137* (1), 289-98.

(4) Ravichandran, K. R.; Taguchi, A. T.; Wei, Y.; Tommos, C.; Nocera, D. G.; Stubbe, J., A >200 meV Uphill Thermodynamic Landscape for Radical Transport in Escherichia coli Ribonucleotide Reductase Determined Using Fluorotyrosine-Substituted Enzymes. *J. Am. Chem. Soc.* **2016,** *138* (41), 13706-13716.

(5) Nick, T. U.; Ravichandran, K. R.; Stubbe, J.; Kasanmascheff, M.; Bennati, M., Spectroscopic Evidence for a H Bond Network at Y356 Located at the Subunit Interface of Active E. coli Ribonucleotide Reductase. *Biochemistry* **2017,** *56* (28), 3647-3656.

(6) Mims, W. B., Pulsed endor experiments. *Proc. R. Soc. London, Ser. A* **1965,** *283* (1395), 452-457.

(7) Bruggemann, W.; Niklas, J. R., Stochastic ENDOR. *J. Magn. Reson.* **1994,** *108* (1), 25-29.

(8) Epel, B.; Arieli, D.; Baute, D.; Goldfarb, D., Improving W-band pulsed ENDOR sensitivity—random acquisition and pulsed special TRIPLE. *J. Magn. Reson.* **2003,** *164* (1), 78-83.

(9) Rizzato, R.; Bennati, M., Enhanced sensitivity of electron-nuclear double resonance (ENDOR) by cross polarisation and relaxation. *Phys. Chem. Chem. Phys.* **2014,** *16* (17), 7681-5.

(10) Zanker, P. P.; Jeschke, G.; Goldfarb, D., Distance measurements between paramagnetic centers and a planar object by matrix Mims electron nuclear double resonance. *J. Chem. Phys.* **2005,** *122* (2), 024515.

(11) Doan, P. E.; Lees, N. S.; Shanmugam, M.; Hoffman, B. M., Simulating suppression effects in Pulsed ENDOR, and the 'hole in the middle' of Mims and Davies ENDOR Spectra. *Appl. Magn. Reson.* **2010,** *37* (1-4), 763-779.

(12) Stoll, S.; Britt, R. D., General and efficient simulation of pulse EPR spectra. *Phys. Chem. Chem. Phys.* **2009,** *11* (31), 6614-25.

(13) Stoll, S.; Schweiger, A., EasySpin, a comprehensive software package for spectral simulation and analysis in EPR. *J. Magn. Reson.* **2006,** *178* (1), 42-55.

(14) Edmonds, D. T.; Zussman, A., Pure quadrupole resonance of 17O in ice. *Phys. Lett. A* **1972,** *41* (2), 167-169.

(15) Neese, F., The ORCA program system. *Wiley Interdiscip. Rev.: Comput. Mol. Sci.* **2012,** *2* (1), 73-78.

(16) Perdew, J. P., Density-functional approximation for the correlation energy of the inhomogeneous electron gas. *Phys. Rev. B* **1986,** *33* (12), 8822-8824.

(17) Becke, A. D., Density-functional exchange-energy approximation with correct asymptotic behavior. *Phys. Rev. A* **1988,** *38* (6), 3098-3100.

(18) Schäfer, A.; Huber, C.; Ahlrichs, R., Fully optimized contracted Gaussian basis sets of triple zeta valence quality for atoms Li to Kr. *J. Chem. Phys.* **1994,** *100* (8), 5829-5835.

(19) Weigend, F.; Ahlrichs, R., Balanced basis sets of split valence, triple zeta valence and quadruple zeta valence quality for H to Rn: Design and assessment of accuracy. *Phys. Chem. Chem. Phys.* **2005,** *7* (18), 3297-305.

(20) Neese, F.; Wennmohs, F.; Hansen, A.; Becker, U., Efficient, approximate and parallel Hartree–Fock and hybrid DFT calculations. A 'chain-of-spheres' algorithm for the Hartree–Fock exchange. *Chem. Phys.* **2009,** *356* (1-3), 98-109.

(21) Grimme, S.; Antony, J.; Ehrlich, S.; Krieg, H., A consistent and accurate ab initio parametrization of density functional dispersion correction (DFT-D) for the 94 elements H-Pu. *J. Chem. Phys.* **2010,** *132* (15), 154104.
(22) Grimme, S.; Ehrlich, S.; Goerigk, L., Effect of the damping function in dispersion corrected density functional theory. *J. Comput. Chem.* **2011,** *32* (7), 1456-65.
(23) Lee, C.; Yang, W.; Parr, R. G., Development of the Colle-Salvetti correlation-energy formula into a functional of the electron density. *Phys. Rev. B* **1988,** *37* (2), 785-789.
(24) Becke, A. D., Density-functional thermochemistry. III. The role of exact exchange. *J. Chem. Phys.* **1993,** *98* (7), 5648-5652.
(25) Barone, V., Structure, Magnetic Properties and Reactivities of Open-Shell Species from Density Functional and Self-Consistent Hybrid Methods. In *Recent Advances in Density Functional Methods, Part1*, 1996; p 287.
(26) Seyedsayamdost, M. R.; Chan, C. T.; Mugnaini, V.; Stubbe, J.; Bennati, M., PELDOR spectroscopy with DOPA-beta2 and NH2Y-alpha2s: distance measurements between residues involved in the radical propagation pathway of E. coli ribonucleotide reductase. *J. Am. Chem. Soc.* **2007,** *129* (51), 15748-9.
(27) Ravichandran, K. R.; Minnihan, E. C.; Wei, Y.; Nocera, D. G.; Stubbe, J., Reverse Electron Transfer Completes the Catalytic Cycle in a 2,3,5-Trifluorotyrosine-Substituted Ribonucleotide Reductase. *J. Am. Chem. Soc.* **2015,** *137* (45), 14387-95.
(28) Sahlin, M.; Petersson, L.; Graslund, A.; Ehrenberg, A.; Sjoberg, B. M.; Thelander, L., Magnetic interaction between the tyrosyl free radical and the antiferromagnetically coupled iron center in ribonucleotide reductase. *Biochemistry* **1987,** *26* (17), 5541-8.
(29) Lawrence, C. C.; Bennati, M.; Obias, H. V.; Bar, G.; Griffin, R. G.; Stubbe, J., High-field EPR detection of a disulfide radical anion in the reduction of cytidine 5'-diphosphate by the E441Q R1 mutant of Escherichia coli ribonucleotide reductase. *Proc Natl Acad Sci U S A* **1999,** *96* (16), 8979-84.
(30) Stoll, S.; Goldfarb, D., EPR Interactions - Nuclear Quadrupole Couplings. In *eMagRes*, 2017; pp 495-510.
(31) Löwdin, P. O., On the Non-Orthogonality Problem Connected with the Use of Atomic Wave Functions in the Theory of Molecules and Crystals. *J. Chem. Phys.* **1950,** *18* (3), 365-375.
(32) Tiesinga, E.; Mohr, P. J.; Newell, D. B.; Taylor, B. N. CODATA Recommended Values of the Fundamental Physical Constants: 2018. https://physics.nist.gov/cuu/Constants/index.html.
(33) Lin, Q.; Parker, M. J.; Taguchi, A. T.; Ravichandran, K.; Kim, A.; Kang, G.; Shao, J.; Drennan, C. L.; Stubbe, J., Glutamate 52-β at the α/β subunit interface of Escherichia coli class Ia ribonucleotide reductase is essential for conformational gating of radical transfer. *J. Biol. Chem.* **2017,** *292* (22), 9229-9239.
(34) Kang, G.; Taguchi, A. T.; Stubbe, J.; Drennan, C. L., Structure of a trapped radical transfer pathway within a ribonucleotide reductase holocomplex. *Science* **2020,** *368* (6489), 424-427.
(35) Argirevic, T.; Riplinger, C.; Stubbe, J.; Neese, F.; Bennati, M., ENDOR spectroscopy and DFT calculations: evidence for the hydrogen-bond network within alpha2 in the PCET of E. coli ribonucleotide reductase. *J. Am. Chem. Soc.* **2012,** *134* (42), 17661-70.
